# PKR Protects the Major Catalytic Subunit of PKA Cpk1 from FgBlm10-Mediated Proteasome Degradation in *Fusarium graminearum*

**DOI:** 10.3390/ijms231810208

**Published:** 2022-09-06

**Authors:** Chen Gong, Daiying Xu, Daiyuan Sun, Xue Zhang

**Affiliations:** State Key Laboratory of Crop Stress Biology for Arid Areas, College of Plant Protection, Northwest A&F University, Yangling 712100, China

**Keywords:** FgBlm10, 26S proteasome, PKA, degradation, *Fusarium graminearum*

## Abstract

For optimal proteolytic function, the proteasome core (CP or 20S) must associate with activators. The cAMP-PKA pathway is reported to affect the activity of the proteasome in humans. However, the relationship between the proteasome and PKA is not well characterized. Our results showed that the major catalytic subunit Cpk1 was degraded without the protection of Pkr. Eleven (out of 67) *pkr* suppressors had FgBlm10 C-terminal truncation, one suppressor had an amino acid change mutation in the *PRE*6 ortholog (FGRRES_07282), and one in the *PRE*5 ortholog (FGRRES_05222). These mutations rescued the defects in growth and conidial morphology, Cpk1 stability, and PKA activities in the *pkr* mutant. The interaction of FgBlm10 with FgPre5 and FgPre6 were detected by co-immunoprecipitation, and the essential elements for their interaction were characterized, including the FgBlm10 C-terminus, amino acid D82 of FgPre6 and K62 of FgPre5. Additional FgBlm10-interacting proteins were identified in the wild type and *pkr* mutant, suggesting that PKA regulates the preference of FgBlm10-mediated proteasome assembly. In addition, PKA indirectly affected the phosphorylation of FgBlm10, and its localization in the nucleus. The truncation of the FgBlm10 C terminus also enhanced nuclear import and bleomycin resistance, suggesting its role in proteasome assembly at DNA damage sites. Collectively, our data demonstrated that regulation between PKA and proteasome degradation is critical for the vegetative growth of *F. graminearum*.

## 1. Introduction

The ubiquitin-26S proteasome degradation system (UPS) is essential for the degradation of dysfunctional and short-lived regulatory proteins of all eukaryotic organisms [1]. The 26S proteasome consists of two modules: a 20S core particle (CP) and a proteasome activator (PA) [2]. The CP is a barrel-shaped structure composed of four stacked heptameric rings, with two α-type end rings and two central β-type rings [1]. The β rings consist of seven β-subunits each, which form a sequestered proteolytic chamber [3]. The α-rings consist of seven α-subunits each, which form a kind of gate and restrict the access of polypeptides to the catalytic chamber unless regulatory complexes are bound to the CP [4]. Three classes of PAs were identified in eukaryotes: the ATP-independent Blm10/PA200 [5], 11S/PA28/PA26 (not present in yeast) [6,7], and the ATP-dependent 19S/PA700/RP activators [4,8,9], all of which inserted their C terminus into α-subunit pockets to enable substrate access in the similar manner [4,10,11]. The two ends of the barrel-shaped 20S proteasome can bind either the same or different regulatory particles forming homogeneous or hybrid proteasomes, respectively [10,11]. In general terms, 19S/RP associated with 20S CP degrades the majority of substrates. The other activators have the potential to function individually as competitors to prevent RP binding [12,13].

Blm10/PA200 is a conserved monomeric proteasome activator with multiple HEAT-like repeats, which form α-helical structures to stabilize a partially open conformation with its C-terminal HbYX element insertion into α5/α6 pockets [14]. Blm10-CP has been reported to be involved in the degradation of short peptides [15], N-terminal Huntingtin fragments (N-Htt) [10], unstructured proteins such as tau and Dnm1 [15], and acetylated histones [16,17,18] in a ubiquitin- and ATP-independent manner. In addition, Blm10 appears to be required for the degradation of Sfp1, but a clear mechanism of action is unknown [19]. The exact function of Blm10 has been controversial. Considering that Blm10 is also found in an immature form of CP, a role in CP maturation has also been proposed. However, deletion of Blm10 showed no obvious defect in CP maturation [20,21]. Another possible function of Blm10 is in regulating the localization or transport of CP into the nucleus [22] or proteasome storage granules (PSGs) [23], but the Blm10-dependent import pathway is complementary to the canonical nuclear import pathway [18].

The cAMP-protein kinase A (PKA) signaling pathway is one of the major signal transduction pathways that regulates various differentiation and infection processes in eukaryotes [24,25,26]. Activation of G-protein-coupled receptors (GPCRs) mobilizes compartmentalized pulses of cAMP. The binding of cAMP with the regulatory subunits releases its inhibitory binding with the catalytic subunits, and the released catalytic subunits of PKA activate the downstream targets. Reassociation of catalytic subunits and regulatory subunits terminates the signal. According to kinetic parameters for the PKA regulation and association of catalytic subunits in mammals, only a small fraction of total catalytic subunits are released during stimulations [27,28], and regulatory subunits are expressed at high concentrations in molar excess of catalytic subunits to recapture the catalytic subunits [29,30]. Deletion of the regulatory subunit *PKA RIIβ*, enhances the activity of PKAc subunits, leading to disordered adipose metabolism and neuronal activities in mice [31,32]. Mammals have four R-subunit genes, which support the PKA isozyme switch. The decrease of PKA regulatory subunit IIα and IIβ was suggested to be related to a compensatory increase of PKA-RIα [33]. All fungal species of ascomycetes and basidiomycetes with a known genome sequence have only one R-subunit [34,35]. In filamentous fungi *F. graminearum*, *Aspergillus fumigatus,* and *Colletotrichum lagenarium*, the deletion of *PKR* caused severe defects in growth and conidiation [36,37]. In *C. lagenarium*, PKA activity is not detected in the *pkr1* mutant, which cannot be rescued by the addition of cAMP [37]. In *Dictyostelium* cells, the inhibition of catalytic subunits of PKA were released when cAMP binds to the regulatory subunit PkaR. Deletion of *PKAR* resulted in constitutive activity of PkaC, and and overexpressed PKA-C enabled cell aggregation [38,39]. Regulation of PKA subunit expression by inhibitors of proteasomes suggested proteasome-mediated degradation in neuronal cells [40]. It was reported that cAMP or activation of PKA stimulates 26S proteasome activity and intracellular degradation of misfolded proteins [41]. However, the relationship between these two pathways is unclear.

*Fusarium graminearum* is the major causal agent of Fusarium head blight (FHB), which is one of the most important diseases of various cereal crops worldwide. Under favorable environmental conditions, epidemiological outbreaks of FHB cause severe yield losses and reduced grain quality. *F. graminearum* produces harmful mycotoxins, such as deoxynivalenol (DON) and zearalenone. *F. graminearum* is a homothallic fungus that is amenable to classic and molecular genetic studies. As in other fungal pathogens [24,25], the cAMP-PKA pathway is one of the well-conserved signal transduction pathways in *F. graminearum* that regulates various infection processes [36,42]. The *CPK1* and *CPK2* genes encoding the major and minor catalytic subunits of PKA, respectively, as well as the *PKR* gene encoding the regulatory subunit have been functionally characterized in *F. graminearum* [36,42]. The *pkr* mutant in *F. graminearum* expressed severe defects in growth, differentiation, and virulence. Interestingly, *pkr* deletion mutants of *F. graminearum* were unstable and produced spontaneous suppressor strains that had a faster growth rate. In our studies, we identified 11 of 67 *pkr* suppressors with nonsense mutations in 26S proteasome gate protein FgBlm10, suggesting the genetic interaction between the cAMP-PKA signaling pathway and 26S proteasome.

In this study, we analyzed the type of mutations of *pkr* suppressors in *FgBLM10* and found that all of the suppressors had nonsense mutations resulting in the truncation of the C-terminal region in FgBlm10. We further confirmed the suppression of FgBlm10 by deleting its C-terminal region in the *pkr* mutant. The *FgBLM10*^∆CT1566^ *pkr* and *FgBLM10*^∆CT11^ *pkr* mutants partially rescued the growth and the conidiation defects of *pkr* and produced conidia with normal morphology. Interestingly, Cpk1 proteins were not detectable in the *pkr* mutant. *FgBLM10*^∆CT1566^ *pkr* mutants and *pkr* suppressors with mutations in *FgBLM10, FgPRE5*^K62E^, and *FgPRE6*^D82N^ suppress Cpk1 degradation and protect PKA activity. Furthermore, the C-terminal region of FgBlm10 and the amino acid K62 in FgPre5 and D82 in FgPre6 are essential for their interactions, suggesting that FgPre5, FgPre6, and FgBlm10 may impact the activity of 26S proteasome for degrading Cpk1 proteins in the *pkr* mutant. In addition, PKR regulated the proteins interacting with FgBlm10 and indirectly regulated the phosphorylation of FgBlm10, which may affect the proteasome assembly and the degradation of Cpk1 in *pkr* mutant.

## 2. Results

### 2.1. Identification of Suppressor Mutations in FgBlm10, the Activator of 20S Proteasome

**Figure 1:**
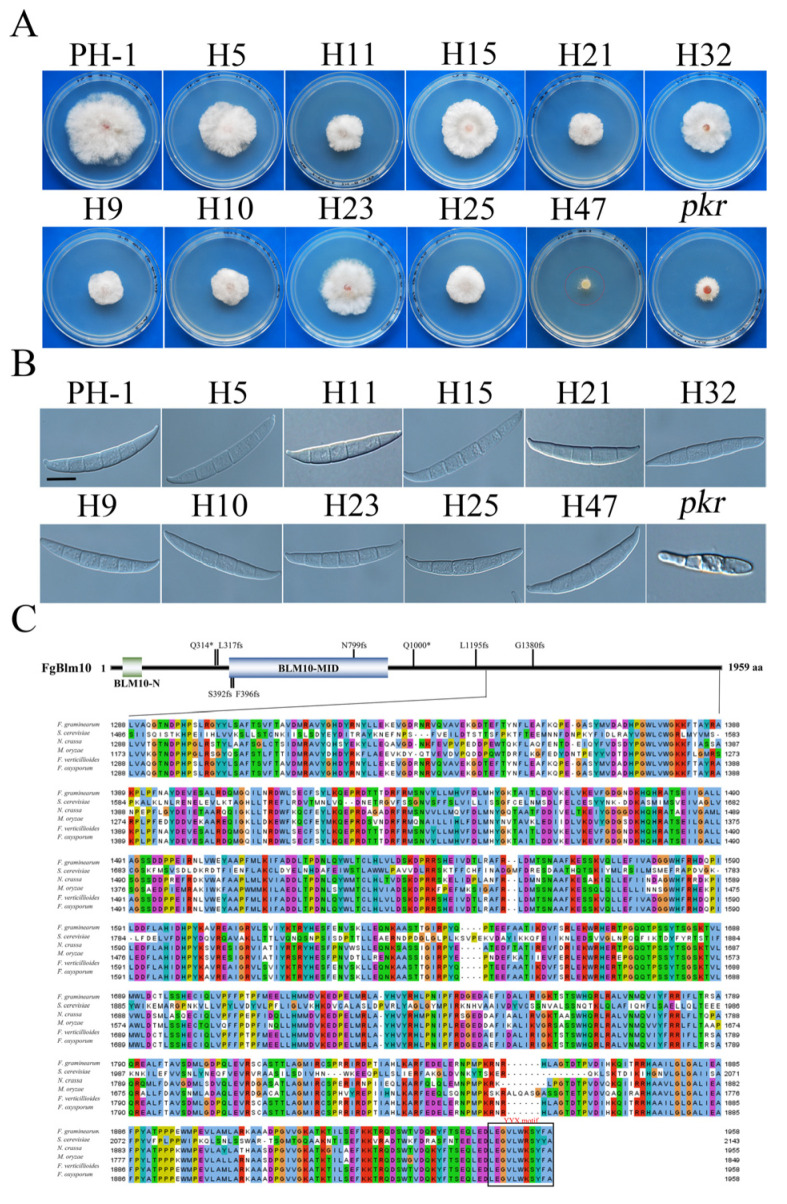
Suppressors of *pkr* with mutations in *FgBLM10* and structural features of FgBlm10. (**A**) Three-day-old PDA cultures of the *pkr* mutant and marked suppressors with mutations in FgBlm10. (**B**) Conidia of the same set of strains from 5-day-old CMC cultures. Bar = 10 µm. (**C**) Schematic drawing of the FgBlm10 protein and alignment of its C-terminal 1566-aa with orthologs from *Fusarium graminearum* (Fg), *Saccharomyces cerevisiae* (Sc), *Neurospora crassa* (Nc), *Magnaporthe oryzae* (Mo), *F. oxysporum* (Fo), and *F. verticillioides* (Fv). *, Stop codon; fs; frameshift mutant.

The *pkr* mutant had severe growth defects and formed colonies with compact aerial hyphae. Sixty-seven spontaneous suppressors of the *pkr* mutant were isolated [36] and 11 suppressor stains had suppressor mutations in *FgBLM10* identified by whole genome sequencing or PCR amplification and sequencing analysis (Table S1). All of the suppressor strains had nonsense mutations (frameshift or stop codon) in *FgBLM10*, partially rescued the growth and conidiation defects of *pkr* mutant, and produced conidia with normal morphology (Figure 1A,B) (Table S2). We categorized them into two types based on their growth rate and mutation sites. Of the five type I suppressors, H5 and H11, caused truncation of the C-terminal 958, and 578 amino acids, respectively. The other three suppressors, H15, H21, and H32, caused truncation of the C-terminal 763 amino acid. However, none of them affected the BLM10-MID domain (Figure 1C). Suppressors H5, H15, and H32 recovered to approximately 68.3% of that of the wild type. However, H11 and H21 recovered only to 70.8% of that of the wild type. The whole genome sequencing analysis of suppressor H11 showed frameshift mutations in both *FgSNT1* and *FgBLM10*, which may explain the reduction in growth rate compared to other Type I suppressors (Table S2). It is possible that H21 also had an additional mutation since we identified only the putative genes in H21 by PCR amplification and sequencing. Five type II suppressors, H9, H10, H23 H25, and H47, had nonsense mutations that disrupted (H10, H13, H23, and H47) or truncated (H9 and H25) the conserved BLM10-MID domain of FgBlm10 (Table 1). All of the type II suppressors restored the growth rate to approximately 64.7% of that of the wild type (Table S2), suggesting a positive role of the BLM10-MID domain in vegetative growth.

Blm10 has been reported as a 20S activator to open the gate of the CP by binding to the α-rings with its C-terminal residues [4,10,11]. Because the common regions of all these suppressors were the C-terminal 578 residues, we aligned these residues of FgBlm10 with its orthologs from yeast and other filamentous fungi. The C-terminal 578 aa were conserved in yeast and other filamentous fungi, and the YYX motif was conserved in all these species, which was reported to be crucial for its acidity [15] (Figure 1C). It is possible the YYX motif plays a suppressive role in the *pkr* mutant.

### 2.2. Deletion of FgBlm10 C-Terminus Partially Rescued Growth and Conidiation Defects of the pkr Mutant

**Figure 2:**
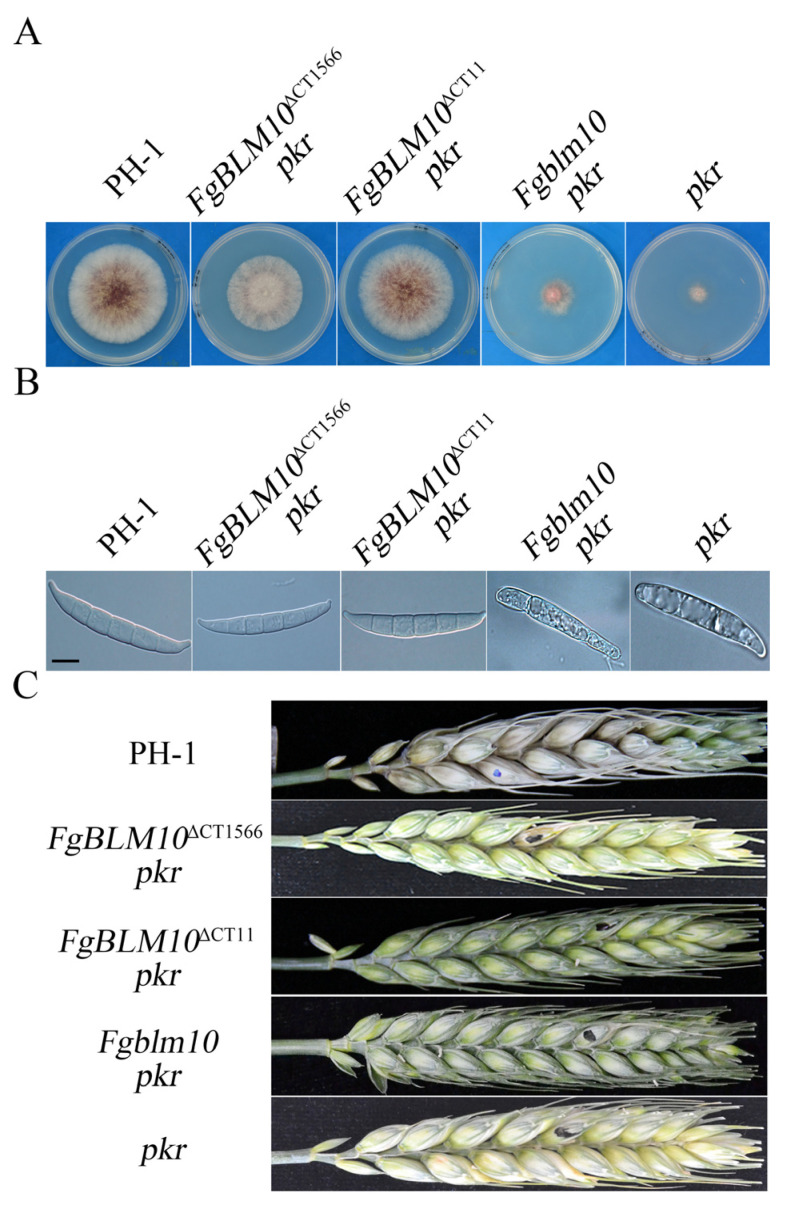
Deletion of the C-terminal region of *FgBlm10* partially rescues the defects of *pkr.* (**A**) Three-day-old PDA cultures of the wild-type strain PH-1 and the *pkr*, *FgBlm10*^∆CT1566^ *pkr*, and *FgBlm10 pkr* mutants. (**B**) Conidia of the same set of strains from 5-day-old CMC cultures. Bar = 10 µm. (**C**) Wheat heads inoculated with the marked strains were examined for head blight symptoms at 14 days post-inoculation (dpi). Black dots mark the inoculated spikelets.

To verify the suppressive effects of C-terminal truncations in *FgBLM10* on the *pkr* mutant, we generated the *FgBLM10*^ΔCT1566^ gene replacement construct in which the C-terminal 1566 amino acid residues were replaced with the geneticin-resistance cassette (Figure S1). In total, three *FgBLM10*^∆CT1566^ *pkr* transformant strains that were resistant to both geneticin and hygromycin were identified. They had identical phenotypes, although data for only one of them, mutant BNP1, are presented below. In comparison with the *pkr* mutant, the *FgBLM10*^∆CT1566^ *pkr* mutant restored the vegetative growth to approximately 75.2% of that of the wild type. Similar to the other *pkr* suppressors, *FgBLM10*^∆CT1566^ *pkr* partially rescued conidiation and produced conidia with normal morphology (Figure 2A,B). However, the *FgBLM10*^∆CT1566^ *pkr* mutant did not rescue the fungal virulence in plant infection (Figure 2C).

To determine the suppressive effects of the conserved YYX motif on the *pkr* mutant, we also deleted the C-terminal 11 amino acid residues of FgBlm10 in *pkr* mutant using the gene replacement approach to generate the *FgBLM10*^ΔCT11^ *pkr* mutant. *FgBLM10*^∆CT11^ *pkr* restored the vegetative growth to approximately 79.1% of that of the wild type, which is 5.1% higher than that of the *FgBLM10*^∆CT1566^ *pkr* mutant (Figure 2A,B). This is consistent with the suppressive effects in *pkr* suppressors.

**Figure 3:**
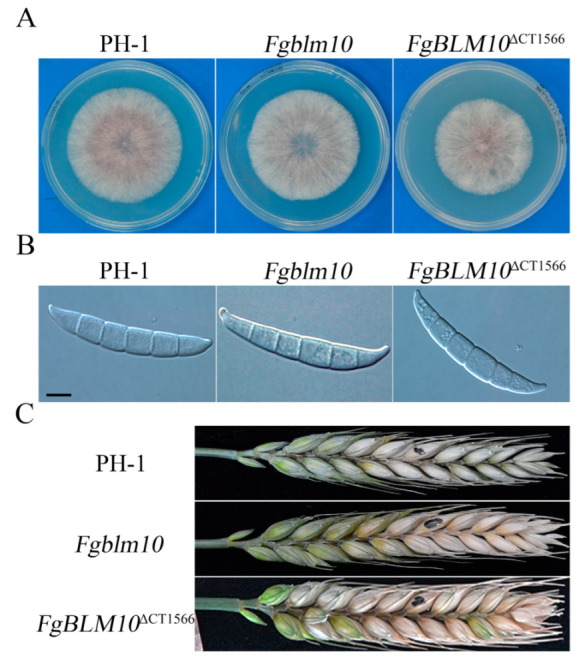
Assays for the phenotypes of the *FgBlm10* and *FgBlm10*^∆CT1566^ mutants. (**A**) Three-day-old PDA cultures of the wild type (PH-1) and the *FgBlm10* and *FgBlm10*^∆CT1566^ mutants. (**B**) Conidia from 5-day-old CMC cultures were examined for morphological defects. Bar = 10 µm. (**C**) Wheat heads inoculated with the marked strains were examined for head blight symptoms at 14 dpi. Black dots mark the inoculated spikelets.

To determine whether deletion of the entire *FgBLM10* can rescue the *pkr1* mutant, we also used the gene replacement approach to generate the *Fgblm10 pkr* double mutant. All three *Fgblm10 pkr* mutant strains (Table 2) were similar to the *pkr* mutant in growth rate and abnormal conidia (Figure 3). Therefore, truncation of its C-terminal region, but not deletion of the entire *FgBlm10*, is suppressive to the *PKR* deletion.

### 2.3. FgBLM10 Is Not Essential for the Growth and Development of F. graminearum

To determine the functions of *Fgblm10* and its C-terminal region, we transformed the *Fgblm10* and *FgBLM10*^∆CT1566^ gene replacement constructs into the wild-type strain PH-1. The resulting gene replacement mutants had no significant changes in vegetative growth (Figure 3A), or conidial morphology (Figure 3B). The conidia production of *FgBlm10* (97.9 ± 15.9 × 10^4^ conidia/mL) is significantly reduced compared to the wild type (247.6 ± 24.7 × 10^4^ conidia/mL), whereas the *FgBlm10*^∆CT1566^ mutant (140.2 ± 23.9 × 10^4^ conidia/mL) produced approximately 1.4-fold more conidia than *FgBlm10* (Table 3). In infection assays with wheat heads, the *Fgblm10* and *FgBLM10*^∆CT1566^ deletion mutants remained pathogenic and caused typical head blight symptoms on inoculated spikelets (Figure 3C). The disease index of the *FgBlm10* mutant (6.5 ± 1.5) was significantly reduced to almost 66.3% of the wild-type strain PH-1 (9.8 ± 2.6), whereas the reduction in virulence (7.7 ± 1.5) of the *FgBLM10*^∆CT1566^ mutant was not as severe as the *Fgblm10* mutant (Table 3). These results indicate that *Fgblm10* is not important for normal hyphal growth but plays a role in conidiation and plant infection. Because deletion of CT1566 had many fewer effects on conidiation and virulence than deletion of the entire gene, the C-terminal region of FgBlm10 likely has a minor role but is not essential for its normal functions in *F. graminearum*.

### 2.4. Two pkr Suppressor Mutations Occur in the α-Ring of the 20S Proteasome

**Figure 4:**
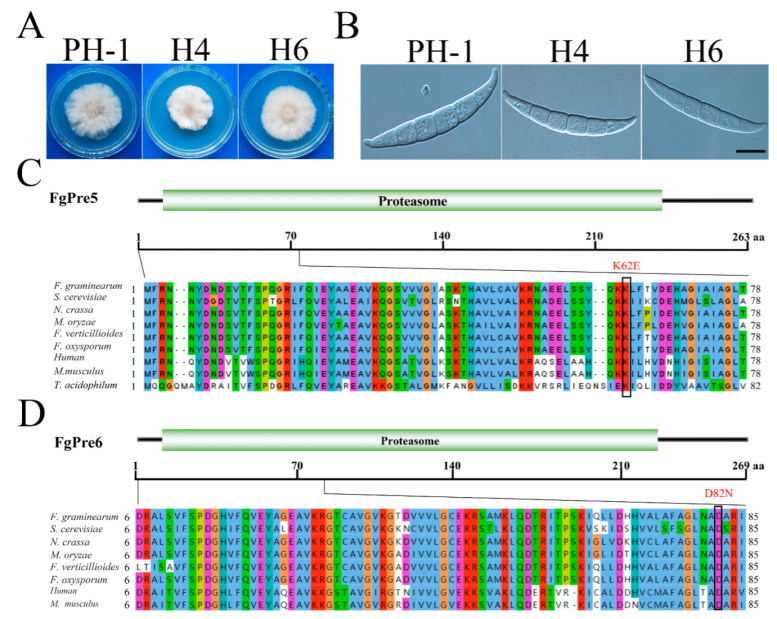
Suppressors of *pkr* with mutations in *FgPRE6* and *FgPRE5*. (**A**) Three-day-old PDA cultures of the *pkr* mutant and marked suppressors with mutations in FgPre5 and FgPre6. (**B**) Conidia of the same set of strains from 5-day-old CMC cultures. Bar = 10 µm. (**C**) Schematic drawing of the FgPre5 protein and alignment of its N-terminal 78-aa with orthologs from *Fusarium graminearum* (Fg), *Saccharomyces cerevisiae* (Sc), *Magnaporthe oryzae* (Mo), *Neurospora crassa* (Nc), *F. oxysporum* (Fo), *Homo sapiens* (Hs), *Mus musculus* (Mm), and *Thermoplasma acidophilum* (Ta). (**D**) Schematic drawing of the FgPre6 protein and alignment of its N-terminal 85-aa with orthologs from *Fusarium graminearum* (Fg), *Saccharomyces cerevisiae* (Sc), *Magnaporthe oryzae* (Mo), *Neurospora crassa* (Nc), *F. oxysporum* (Fo), *Homo sapiens* (Hs), and *Mus musculus* (Mm).

Two suppressors H6 and H4 have mutations in two of the α subunits of the 20S proteasome, identified by whole genome sequencing. Both of them partially rescued the growth defects of the *pkr* mutant, but produced conidia with normal morphology (Figure 4A,B). Although the growth rate of H4 is slightly slower than that of H6, suppressor H4 produced 4.1-fold more conidia than the *pkr* mutant, and H6 did not rescue the conidiation defects of *pkr* (Table S2).

Suppressor strain H6 with the A to G mutation at A325 was identified in FGRRES_05222, which is orthologous to yeast *PRE*5, also known as α6 in 20S CP [44]. The resulting K to E mutation occurred at the K62 residue that is conserved from yeast to human (Figure 4C). In *Thermoplasma acidophilum*, α6Lys66 was reported to form a salt bridge with the C-terminal carboxylate of Blm10 or 11S or 19S activator [10,11,45,46]. Interestingly, K62 in FgPre5 is the same amino acid (α6K66) in *F. graminearum.* Therefore, the amino acid mutation of K62 in FgPre5 may be important to sustain the function of 20S CP or to interact with proteasome activators, which are suppressive to the *pkr* mutant.

Suppressor strain H4 with the G to A mutation at G410 was identified in FGRRES_07282, which is orthologous to yeast *PRE*6, also known as α4 in 20S CP [44]. The resulting D to N mutation occurred at the D82 residue that is conserved from yeast to human (Figure 4D). Asparagine is derivative of aspartic acid, which has an additional carboxyl group. Therefore, the negative charge at specific amino acid D82 of FgPre6 may be important for its function. Blm10 C-terminal residues form β-sheet-like hydrogen bonds with the α5/α6 pocket of 20S CP. Such interaction significantly affects α ring conformation such that the entrance pore in the Blm10-20S complex is partially open [45]. Taken together, our results suggested that the essential amino acids of 20S core particle or C-terminus of activator FgBlm10 or their interactions may be suppressive to the *pkr* mutant.

### 2.5. The C-Terminal Region of FgBlm10 and FgPre5^K62^ Are Important for Their Interactions

**Figure 5:**
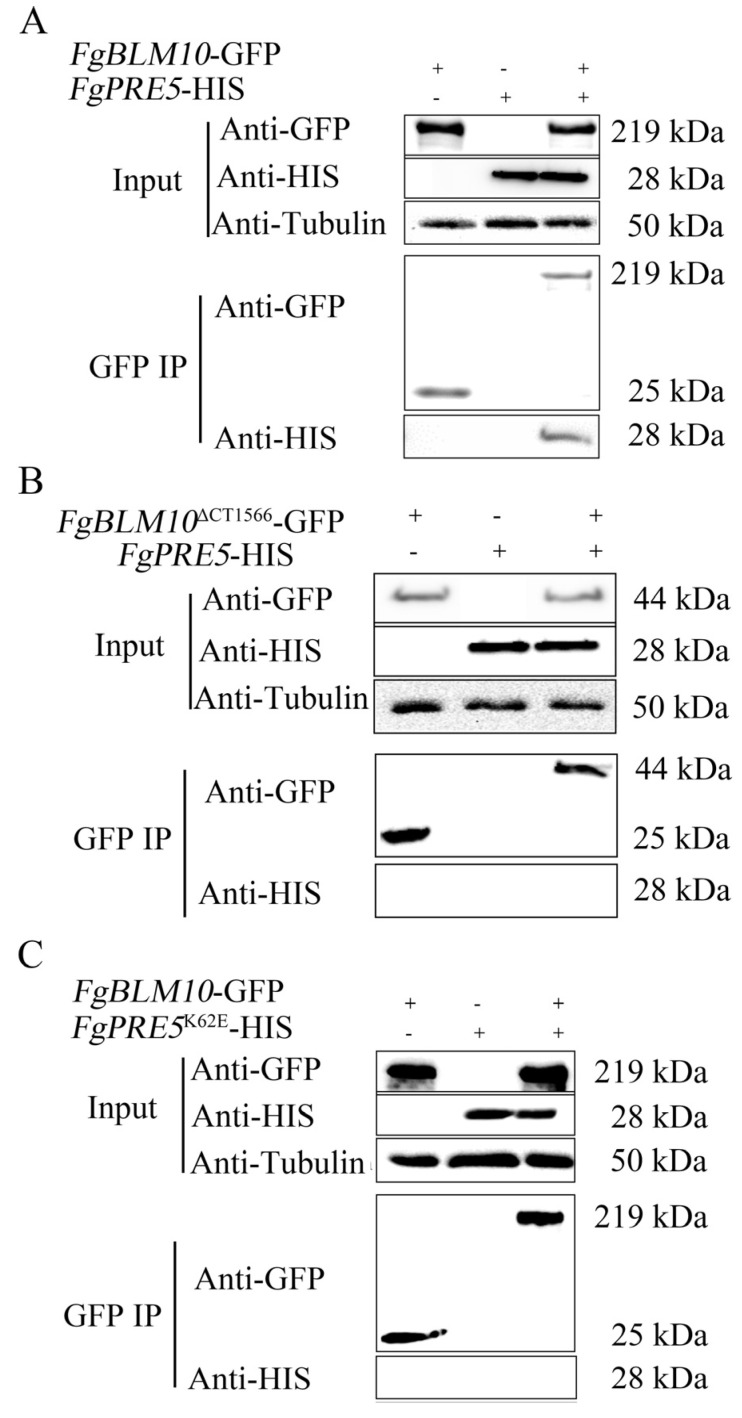
co-IP assays for the interaction of FgPre5 and *FgPRE5*^K52E^ with FgBlm10 and FgBlm10^∆CT1566^. (**A**) Western blots of total proteins (In for input) isolated from transformants expressing the *FgPRE5*-His and *FgBLM10*-GFP constructs and proteins immuno-precipitated (IP) with anti-GFP agarose beads were detected with the anti-GFP and anti-His antibodies. Detection with an anti- tubulin antibody was included as the negative co-IP control. (**B**) Western blots of total proteins and proteins immuno-precipitated with anti-GFP agarose beads of transformant expressing the *FgPRE5*-His and *FgBLM10* ^∆CT1566^-GFP constructs were detected with anti-GFP, anti-His antibodies, or anti- tubulin antibody. (**C**) Western blots of total proteins and proteins immuno-precipitated with anti-GFP agarose beads of transformant expressing the *FgPRE5* ^K52E^-His and *FgBLM10*-GFP constructs were detected with anti-GFP, anti-His antibodies, or anti- tubulin antibody.

To confirm the importance of the C-terminal region of FgBlm10 in its interaction with FgPre5, the *FgBLM10-*GFP and *FgBLM10*^∆CT1566^-GFP constructs were generated and co-transformed into PH-1 with *FgPRE5*-His constructs. The resulting transformants BPR5-1 (Table 1) were confirmed by Western blot analyses for the expression of transforming constructs. In co-IP assays, the FgPre5 band was detected in both total proteins and proteins eluted from anti-GFP agarose beads in the transformant expressing the *FgBLM10-*GFP and *FgPRE5*-His constructs (Figure 5A). However, the FgPre5 band was detected only in total proteins isolated from the transformant expressing *FgBLM10*^∆CT1566^-GPF and *FgPRE5*-His constructs (Figure 5B). These results confirmed that FgPre5 interacts with the full-length FgBlm10 but not FgBlm10^∆^^CT1566^ in *F. graminearum*.

To determine the function of K62 in the FgPre5-FgBlm10 interaction, we generated the *FgPRE5* ^K62E^-His construct and co-transformed it with FgBlm10-GFP. In co-IP assays, the *FgPRE5* ^K62E^-His band was detected only in total proteins from transformant strain BPRK3, indicating that the amino acid mutations in K62E of *FgPRE5* affect its interaction with FgBlm10 (Figure 5C).

### 2.6. The C-Terminal Region of FgBlm10 and FgPre6^D82^ Are Important for Their Interactions

**Figure 6:**
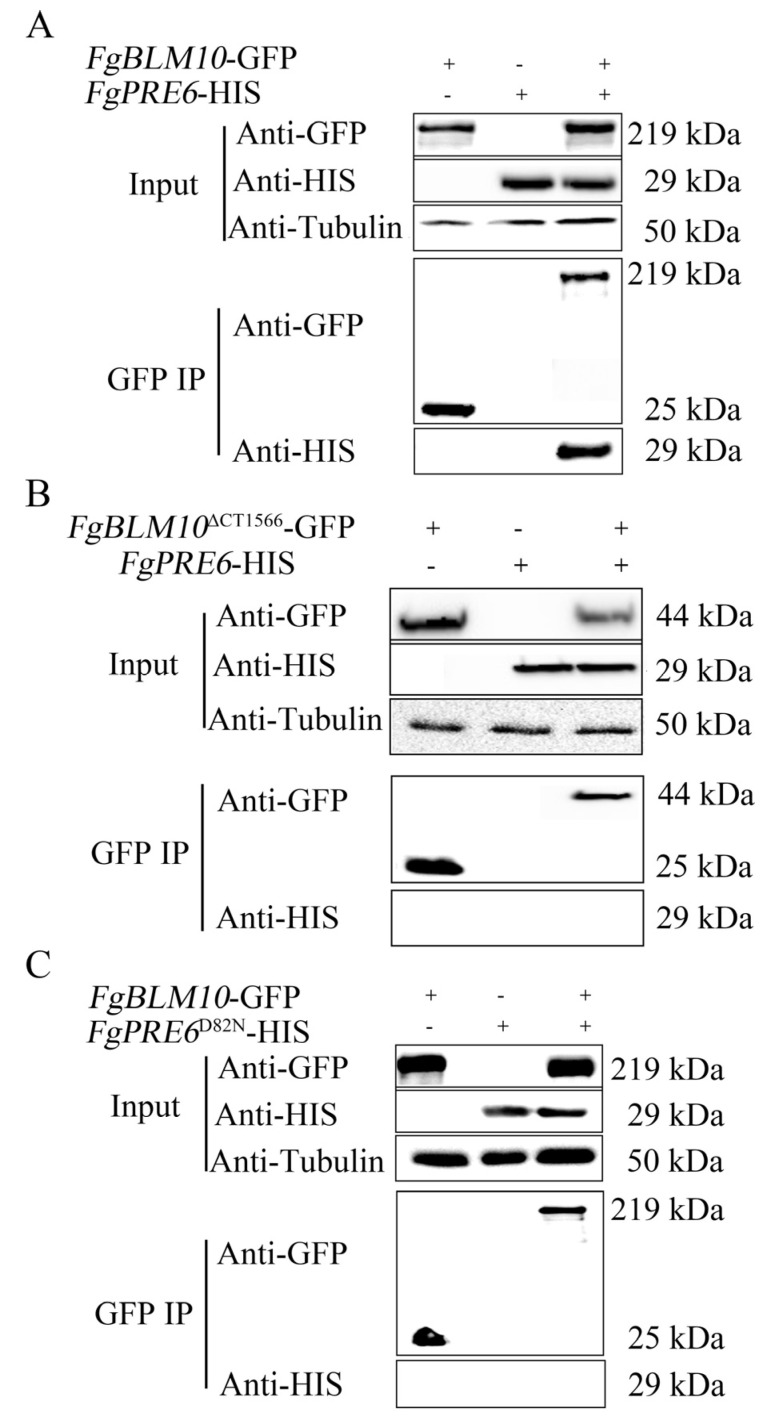
co-IP assays for the interaction of FgPre6 and *FgPRE6*^D82^ with FgBlm10 and FgBlm10^∆CT1566^. (**A**) Western blots of total proteins (In for input) isolated from transformants expressing the *FgPRE6*-His and *FgBLM10*-GFP constructs and proteins immuno-precipitated (IP) with anti-GFP agarose beads were detected with the anti-GFP and anti-His antibodies. Detection with an anti- tubulin antibody was included as the negative co-IP control. (**B**) Western blots of total proteins and proteins immuno-precipitated with anti-GFP agarose beads of transformant expressing the *FgPRE6*-His and *FgBLM10* ^∆CT1566^ -GFP constructs were detected with anti-GFP, anti-His antibodies, or anti- tubulin antibody. (**C**) Western blots of total proteins and proteins immuno-precipitated with anti-GFP agarose beads of transformant expressing the *FgPRE6* ^D82N^-His and *FgBLM10*-GFP constructs were detected with anti-GFP, anti-His antibodies, or anti- tubulin antibody.

Similarly, to determine the interaction of the C-terminus of FgBlm10 with FgPre6, we generated the FgPre6-HIS construct and co-transformed it with *FgBLM10*-GFP and *FgBLM10*^∆CT1566^-GFP. The resulting transformant BPR6-3 (Table 1) was confirmed by Western blot analyses for the expression of transforming constructs. The FgPre6 band was detected in both total proteins and those eluted from anti-GFP agarose beads in the transformant expressing the *FgBLM10-*GFP and *FgPRE6*-His constructs (Figure 6A). However, the FgPre6 band was detected only in total proteins isolated from the transformant expressing *FgBLM10***^∆^**^CT1566^-GFP and *FgPRE6*-His constructs (Figure 6B). These results confirmed that FgPre6 interacts with the full-length FgBlm10 but not FgBlm10^∆CT1566^ in *F. graminearum*.

To determine the function of D82 in FgPre6-FgBlm10 interaction, we generated the *FgPRE6*^D82N^-His construct and co-transformed it with FgBlm10-GFP. In co-IP assays, *FgPRE6*^D82N^ -His band was detected only in total proteins from transformant strain BPRD7, indicating that the amino acid mutations in D82N of *FgPRE5* affect its interaction with FgBlm10 (Figure 6C).

### 2.7. Pkr Is Important for Protecting Cpk1 from Degradation by the 26S Proteasome

**Figure 7:**
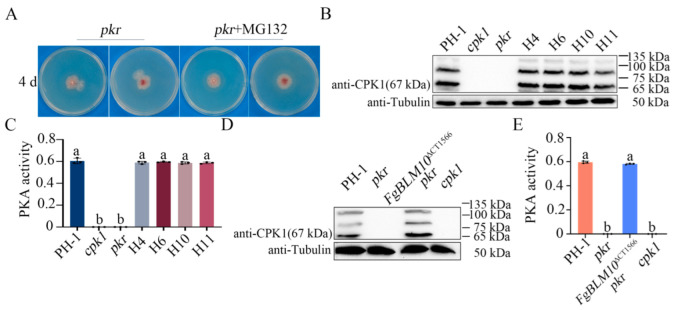
Assays for PKA activities and Cpk1 expression. (**A**) Four-day-old PDA cultures of the *pkr* mutant with or without 50µM MG132. (**B**) Western blots of total proteins isolated from the marked strains and treatment were detected with an anti-FgCpk1 antibody. The expected size of Cpk1 is 67 kDa. Detection with an anti-tubulin antibody was used as the loading control. (**C**) PKA activity was assayed with proteins isolated from hyphae of PH-1, *cpk1*, *pkr*, H4, H6, H10, and H11. (**D**) Western blots of proteins isolated from the marked strains were detected with an anti-FgCpk1 antibody. (**E**) PKA activity was assayed with proteins from the marked strains. Different letters indicate significant differences based on ANOVA analysis followed by Duncan’s multiple range test (*p* = 0.05) in a, b.

Because mutations in the key components of the proteasome, *FgPRE5*, and *FgPRE6*, and its activator *FgBLM10*, suppressed the growth defects of *pkr*, it is possible that *pkr* is stabilized by the inhibition of FgBlm10-mediated proteasome degradation. To test this hypothesis, the *pkr* mutants were cultivated at PDA plates with 26S proteasome inhibitor MG132 for four days. On the 4-day-old PDA plates, the *pkr* mutants produced spontaneous suppressors with a faster growth rate (Figure 7A). However, the spontaneous suppressor was not produced on the 4-day-old PDA plates with 50 µM MG132, suggesting that inhibition of the proteasome may stabilize the *pkr* mutant and prevent the spontaneous mutation of the *pkr* mutant (Figure 7A).

Our study showed that, in the absence of Pkr, Cpk1 proteins were degraded, and the PKA activities of Cpk1 were abolished. When treated with 50 µM MG132, the Cpk1 band and PKA activities can be detected in the *pkr* mutant (Figure S3). Suppressor mutations in components of the 26S proteasome likely affect the 26S proteasome function and Cpk1 degradation. When assayed for Cpk1 degradation with the anti-CPK1 antibody, the 67 kDa Cpk1 band was detected in suppressor strains H10 and H11 with a suppressor mutation in *FgBLM10*, H6 with a mutation in FgPre5, and H5 with a mutation in FgPre6 (Figure 7B). The PKA activity was also detected in all suppressors H4, H6, H10, and H11 (Figure 7C). In addition, we detected the PKA activity and Cpk1 degradation in the *Fgblm10*^∆CT1566^ *pkr* mutant. Similar to the suppressor strain H10, deletion of the C-terminal region of FgBlm10 recovered the PKA activities and prevented degradation of Cpk1 in the *pkr* mutant (Figure 7D,E). These results suggested that the major PKA catalytic subunit Cpk1 is protected by its regulatory subunit Pkr in the wild type, and Cpk1 degradation was mediated by the proteasome activator FgBlm10 and its α-ring subunits FgPre5 and FgPre6 in the absence of Pkr.

### 2.8. FgBlm10 Differentially Interacts with the RP and Core Components of the 26S Proteasome in the pkr Mutant and Wild Type

To identify proteins differentially interacting with FgBlm10 in the background of the WT and *pkr* mutant, we generated the *FgBLM10*-GFP construct and transformed it into PH-1 and *pkr* mutant, respectively. Total proteins were isolated from the resulting *FgBLM10*-GFP/PH-1 and *FgBLM10*-GFP/*pkr* transformants and used for affinity purification with anti-GFP agarose beads. Proteins were identified by mass spectrometry (MS) analysis after trypsin digestion, as described in previous studies [26]. Blm10 has been reported as a 20S activator to open the gate of the CP by binding to the α-rings with its C-terminal residue. Based on the MS results, FgBlm10 interacted with core particles of the 26S proteasome PRE6, PRE7, PRE8, and PRE9 as expected (Table 4). The interaction of FgBlm10 with FgPre6 was confirmed by IP assay (Figure 6). Compared to the interaction of FgBlm10 with FgPre6, the interaction between FgBlm10 and FgPre5 is weaker based on the co-IP assay and, therefore, FgPre5 was not co-purified in the IP-MS assay.

In yeast, Blm10 in unperturbed lysates is predominantly bound to form a Blm10-CP-RP hybrid proteasome, although the substrate of this hybrid proteasome is unclear [47]. In *F. graminearum* wild-type strain PH-1, FgBlm10 also interacted with most components of the RP activator, including the structural subunits RPN12, RPN2, RPN3, RPN5, RPN6, RPN7, RPN8, and RPN9, and ATPase subunits RPT1, RPT2, RPT3, RPT4, RPT5, and RPT6 (Table 4) [48,49]. However, FgBlm10 only interacted with FgPre6 and four of the RP activator components, RPT6, RPT4, RPN9, and RPN7 in the *pkr* mutant. The number of RPT6, RPT4, RPN9, and RPN7 peptides identified by MS analysis (Table 4) was significantly lower in the *pkr* mutant than in the wild-type background, suggesting a weaker association of FgBlm10 with the RP-20S complex. In yeast, increased expression of Blm10 outcompeted RP for CP binding, and overexpression of BLM10 almost eliminated the presence of RP-CP complexes [13]. Accordingly, we determined the expression level of FgBLM10 in the wild type and *pkr* mutant by qRT-PCR. The results showed that the transcriptional level of FgBlm10 in the *pkr* mutant was increased to 4-fold higher compared to the wild type (Figure S4). It is likely that PKA affects the preference of FgBlm10 in the association as RP-CP-FgBlm10 but not FgBlm10-CP species, although its mechanism is unclear.

### 2.9. PKA Indirectly Affects the Phosphorylation Level of FgBlm10

To determine the physical interaction between FgBlm10 with PKA, we generated the prey constructs with Pkr and Cpk1 and co-transformed them with the bait constructs of FgBlm10. Neither yeast transformant expressing the prey and FgBlm10 bait was able to grow on SD-Trp-Leu-His medium, indicating that FgBlm10 does not directly interact with the Pkr or Cpk1 (Figure S5). Consistent with that observation, FgBlm10 was not co-purified with either of the components of PKA in the IP-MS assay.

To determine whether the FgBlm10 affects the PKA activity, we determined the transcriptional level of Cpk1 and the PKA activity. The results showed that neither the *Fgblm10* nor the *FgBLM10*^∆CT1566^ mutant affected the expression of Cpk1. However, the PKA activity was slightly upregulated in *Fgblm10*, compared to the wild type and *FgBLM10*^∆CT1566^ mutant (Figure S2). These results further suggested that FgBlm10 is not essential for fungal development or PKA activity in the presence of Pkr.

To further investigate the relationship between PKA and FgBlm10, we isolated total proteins from the *FgBLM10*-GFP/PH-1 and *FgBLM10*-GFP/*pkr* transformants and incubated them with anti-GFP agarose beads. Proteins eluted from anti-GFP beads were treated with trypsin and enriched for phosphopeptides with PolyMac, as described [50]. The resulting phosphopeptides were analyzed by MALDI-TOF/TOFMS [51]. In total, we identified 17 phosphorylation sites on FgBlm10 in wild-type transformants, but only four phosphorylation sites on FgBlm10 in the *pkr* transformants (Figure S6). The differentially phosphorylated amino acids are scattered across the poorly conserved N-terminal region, conserved Blm10-MID domain, and C-terminal region (Figure S6). Although truncation of the conserved C-terminal region of FgBlm10 led to a gain-of-function in *pkr* mutant, the full-length deletion of *FgBLM10* did not rescue the defects of *pkr*. One possible explanation is that the non-phosphorylated N-terminus of FgBlm10 still physically interacts with the proteasome or other factors, thus attenuating their assembly as RP-20S-RP proteasome to degrade catalytic subunits of PKA.

### 2.10. The Nuclear Localization of FgBlm10 Is Regulated by Its C-Terminus and PKA Activity

**Figure 8:**
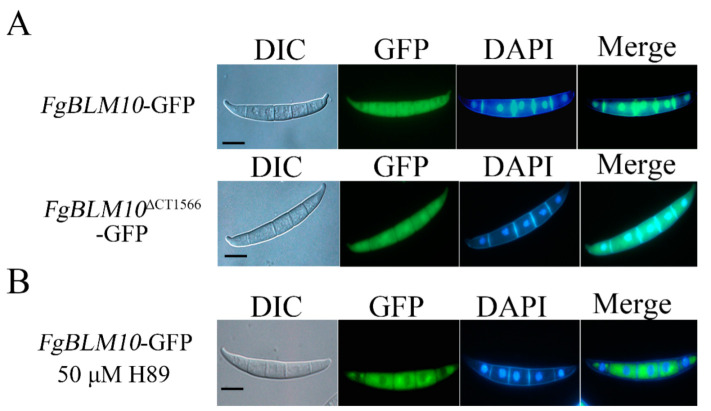
Subcellular localization of *FgBLM10*-GFP and *FgBLM10* ^∆CT1566^ -GFP fusion proteins. (**A**) Conidia of the *FgBLM10*-GFP transformant were examined by differential interference contrast (DIC) and epifluorescence microscopy. Green fluorescent protein (GFP) signals were observed in the nucleus and cytoplasm of conidia. Bar = 10 µm. Nuclei were stained with DAPI (4′,6-diamidino-2-phenylindole). (**B**) Conidia of the *FgBLM10*-GFP transformant treated with 50 µM H89 were examined by differential interference contrast (DIC) and epifluorescence microscopy. Green fluorescent protein (GFP) signals were observed in the nucleus and cytoplasm of conidia. Bar = 10 µm.

The function of the proteasome is also regulated by its subcellular localization [52]. The 20S proteasomes have been shown to diffuse freely between cyto- and nucleoplasm in yeast and mammals [23]. Blm10 transported CP, pre-holo-CP, and CP precursor complexes into the nucleus exiting from quiescence as an alternative import receptor of the canonical β karyopherins/importin [52,53]. To determine the localization of FgBlm10, we transformed *FgBLM10*-GFP and *FgBLM10***^∆^**^CT1566^-GFP constructs into PH-1. We used 4, 6-diamidino-2-phenylindole (DAPI) to label nuclear DNA. FgBlm10-GFP signals were uniformly distributed in the cytoplasm and nucleus, while *FgBLM10***^∆^**^CT1566^-GFP was detected uniformly throughout the cytoplasm, but *FgBLM10***^∆^**^CT1566^-GFP signals were stronger in the nucleus compared to the cytoplasm (Figure 8A). In yeast, Blm10 is predominantly localized to the nucleus, and deletion of the conserved C-terminal 339 aa led to a redistribution of the protein to the cytoplasm [47,54]. However, deletion of the FgBlm10 terminus led to a redistribution of the protein to the nucleus, suggesting that this region is suppressive for FgBlm10 nuclear import. To determine the auto-inhibitory role of the FgBlm10 C terminus, we constructed the FgBlm10^CT^ bait construct and FgBlm10^MT^ and FgBlm10^M^ prey constructs. However, FgBlm10^CT^ did not interact with either the N-terminal or middle region of FgBlm10 (Figure S7).

It is possible that FgBlm10 is transported into the nucleus by nucleoporin. Based on our MS data, FgBlm10 interacts with multiple nucleoporins or nuclear pore proteins in the wild type. However, none of FgBlm10-interacting nucleoporins was detected in the *pkr* mutant (Table S3). Using the PKA inhibitor H89, we further evaluated the effect of cAMP-PKA on the localization of FgBlm10-GFP and found that its localization to nucleus was reduced (Figure 8B). These results suggested the inhibition of cAMP-PKA negatively regulate the nuclear import of FgBlm10, which was consistent with the lack of FgBlm10-interacting nucleoporins or nuclear pore proteins in *pkr* mutant.

### 2.11. Deletion of the Conserved FgBlm10 C Terminus Results in Bleomycin Resistance

**Figure 9:**
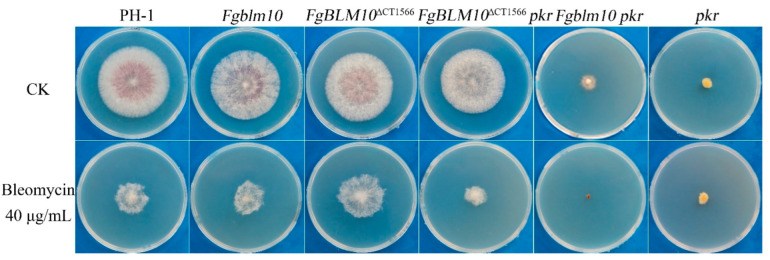
Deletion of the C-terminus of *FgBLM10* enhances the resistance to bleomycin. Three-day-old PDA cultures of the wild-type PH-1, *pkr* mutant, *Fgblm10* mutant, *FgBLM10*^∆CT1566^ mutant, *Fgblm10 pkr* double mutant, and *FgBLM10*^∆CT1566^ *pkr* double mutant in the presence or absence of 40 µg/mL bleomycin.

BLM10 was initially identified as an extragenic suppressor of *blm3-1* under stress induced by the chemotherapeutic drug bleomycin [20]. Blm10 and PA200 were suggested to function in protecting cells from bleomycin-induced DNA damage as a proteasome complex [5,55]. On PDA containing 40 µM bleomycin, the growth rate of the *Fgblm10* mutant (4.5 ± 0.3 mm/day) was slightly increased compared to that of the wild type (4.1 ± 0.1 mm/day), while the *FgBLM10*^∆CT1566^ (5.4 ± 0.1 mm/day) grew 32% faster than wild type. In the *pkr* mutant background, deletion of *FgBLM10* caused higher bleomycin sensitivity than expressed by the *pkr* mutant, while the growth rate of *FgBLM10*^∆CT1566^ *pkr* (2.8 ± 0.2 mm/day) recovered to 68% of that of the wild type (Figure 9, Table 5). These results indicated that deletion of *FgBLM10* did not cause significant sensitivity to bleomycin in the presence of *pkr*. In contrast, deletion of a C-terminal domain of Blm10 compromises the survival of cells exposed to bleomycin in the presence or absence of *pkr*. Based on our MS data, FgBlm10 interacts with multiple DNA damage repair proteins in the wild type [56]. However, none of these DNA damage repair proteins were detected in the *pkr* mutant (Table S3). As described above, FgBlm10 nuclear import is enhanced upon deletion of its C terminus. A possible explanation is that a nuclear-localized *FgBLM10*^∆CT1566^ physically interacts with either the proteasome or other factors involved in DNA repair, thus enhancing their recruitment to the sites of double-strand breaks (DSBs).

## 3. Discussion

PKA holoenzyme consists of two regulatory subunits and two catalytic subunits. Both subunits are important in the growth and virulence of plant pathogens [36,57,58,59]. In previous studies, the regulatory subunit of PKA was considered a selective inhibitor of the catalytic subunits, which were released and activated by cAMP binding to the regulatory subunit in yeast and human [60,61]. However, our study indicated that binding with Pkr regulatory subunits is important for protecting Cpk1 proteins from degradation by the 26S proteasome in *F. graminearum*. Here, we report for the first time that FgBlm10, the ortholog of yeast Blm10 or mammalian PA200, associates with FgPre5 and FgPre6 and plays a role in PKA catalytic subunits degradation in the absence of Pkr. The inhibition of the proteasome by MG132 or dysfunction of the FgBlm10-CP proteasome prevents the degradation of the major catalytic subunits Cpk1 in the *pkr* mutant. PKA regulated the interactions between FgBlm10 with CP and RP to form the RP-CP-FgBlm10 hybrid proteasome in the presence of Pkr, while FgBlm10 prefers to interact with CP in the absence of Pkr. Moreover, the C-terminus of FgBlm10 is involved in its interaction with 20S CP α subunits, FgPre6 and FgPre5, and regulates the localization of FgBlm10 in the nucleus. The formation of the proteasome is consistent with the degradation of Cpk1 in the wild type (RP-CP-FgBlm10), *pkr* mutant (FgBlm10-CP), and *pkr* suppressors (mutations in FgBlm10 and α subunits).

The 20S proteasome (CP) is highly conserved in various eukaryotes and consists of seven outer alpha and seven inner beta subunits (α7β7β7α7). All of the subunits are essential for cellular survival [62,63]. In our study, we found two *pkr* suppressors with mutations in the α-ring of 20S proteasome, one in *PRE*6 ortholog (FGRRES_07282) and the other one in *PRE*5 ortholog (FGRRES_05222). To verify the mutations in FgPre5 and FgPre6, we attempted to delete the *FgPRE5* and *FgPRE6* genes by several independent procedures but those attempts were unsuccessful. It is possible that *FgPRE5* and *FgPRE6* are essential genes in *F. graminearum*. There are only 263 aa in FgPre5 and 269aa in FgPre6, and their sequences are well conserved (Figure S6). It is possible that most of the conserved amino acids play an essential role in cell viability, whereas the mutations of the non-essential amino acids cannot rescue the growth defects of the *pkr* mutant. Although no additional suppressor mutations were identified in FgPre5 and FgPre6 in *pkr* suppressors, the two mutations in α subunits of CP suggested that the components of CP are important for Cpk1 degradation. Interestingly, the Mgp1 (Pre6/Alpha 4) and Mgp5 (Pre5/Alpha 6) were identified as the proteins that are highly induced during appressorium formation in *M. oryzae* [64]. Meanwhile, cAMP-PKA is essential for appressorium turgor generation in *M. oryzae* [26]. In our study, *FgBLM10*^ΔCT1566^ *pkr* restores glycogen accumulation in *pkr* mutant (Figure S8). Bortezomib, a selective inhibitor of the 26S proteasome, induced the proteasome dysfunction, which mediates obesity-induced endoplasmic reticulum stress [65]. It is possible that Blm10-mediated 20S proteasome degradation plays a general role in glycogen and storage proteins accumulation upon the regulation of the PKA pathway in fungi, and plays a role in cell response to stresses in other eukaryotes.

Nonsense mutations (frameshift or stop codon) were detected in 11 of 67 suppressor strains of *FgBLM10*, which not only caused deletion of C-terminal regions, but also disrupted or truncated the conserved BLM10-MID domain of FgBlm10. Although these 11 *pkr* suppressors had a deletion at seven different sites, three of them—H15, H21, and H32— had the same deletion of T^3523^, two of them—H9, and H5—had the T to C substitution at C^986^ and C^3044^, no specific sequence features were found in the flanking DNA sequences of the mutation sites. The overall sequence of Blm10 is well conserved in yeast and other fungi, in which the C-terminal YYX motif is identical (Figure S6). The truncation of the C-terminal as short as 11 residues (CT 11) is suppressive to the *pkr* mutant, suggesting an important and evolutionarily conserved biological role of the C-terminal region. We also assayed the truncation of C-terminal 1566 aa, which disrupted the BLM10-MID and C terminal conserved region. *FgBLM10*^∆CT11^ *pkr* grew faster than *FgBLM10*^∆CT1566^ *pkr*, although none of them fully rescued the growth defects of *pkr* mutant. These results suggested that the BLM10-MID domain of FgBlm10 played a positive role in vegetative growth (Figure 3). The full-length deletion of FgBlm10 cannot rescue the defects of *pkr* mutant, whereas the *Fgblm10* mutant grew faster than *FgBLM10*^∆CT1566^, the N-terminal region of FgBlm10 is necessary to rescue the defects in *pkr*, but suppressive in the growth rate in the presence of Pkr.

In yeast, deletion of BLM10 is not sufficient to produce a global defect in proteasome activity [20,21] but becomes essential in *blm10 rpn4* double deletion with improper proteasome subunits expression [66]. In our study, the deletion of FgBlm10 or its C-terminal mutation, likewise, did not significantly affect fungal growth and development in the presence of Pkr, but FgBlm10 C terminus truncation rescued the growth and conidiation defects in the *pkr* mutant. It is possible that *FgBLM10*^ΔCT^ also becomes essential when the proteasome is not properly phosphorylated by PKA. Previous studies showed that Blm10/PA200 is recruited to the sites of DNA repair and involved in repair mechanisms [5,55]. Truncation of the conserved C-terminus enhanced its localization to nuclear and bleomycin resistance, suggesting its role in DNA damage repair. These observations provided an explanation for the ability of *FgBLM10*^ΔCT1566^ *pkr* to rescue the cell viability defects in the *pkr* mutant [36] (Figure S8). In *Dictyostelium* cells, the proteasome activator PA28/REG is also localized to the nucleus and stimulates the activities of the 20S proteasome in an early unique nuclear degradation pathway [7]. In our previous study, Pkr was suggested to be involved in the regulation of autophagy during hyphal growth [36]. In *D. discoideum,* the components of 19S RP of proteasome, PSMD1, and PSMD2, interacted with the core autophagesomal protein Atg16, which function as the upstream of the ubiquitin-proteasome system [67,68,69]. Therefore, it is also possible that FgBlm10 plays a similar role in interacting with the autophagy pathway.

Previous studies showed that monomeric Blm10 interacts exclusively with only one α subunit, which is located at the α5/α6 pocket [4]. Its C-terminal carboxylate forms a salt bridge with the conserved α6Lys66, whereas a side chain of the penultimate tyrosine interacts with α5Gly19, prompting translocation of the α5Pro17 reverse turn to open the entrance pore [45,46]. In our study, C-terminal truncation in multiple *pkr* suppressors and *FgBLM10*^∆CT1566^ *pkr* mutant partially rescued the defects of vegetative growth, conidial morphology, and conidiation in the *pkr* mutant. In addition, the C-terminal region of FgBlm10 is involved in its interaction with two α subunits of the core proteasome (Figure 5 and Figure 6). H6 suppressor strains had the K62E mutation in FgPre5 (α6Lys62), which is the same amino acid that interacts with Blm10 in α pocket of the 20S core proteasome. Our study further proved that FgBlm10 interacts with FgPre5 instead of FgPre5^K62E^ (Figure 5C). H4 suppressor strains had D82N mutations in FgPre6, and the additional carboxyl group of glutamic acid was involved in its interaction with FgBlm10 (Figure 6C). Importantly, our study confirmed the function of FgPre5Lys62 (α6Lys62) and identified FgPre6Asp82 (α4Asp82) as another important amino acid for the function of the 20S core particle or the interaction with regulatory particles, including FgBlm10. It is noteworthy that the K62E mutation in FgPre5, D82N mutations in FgPre6, or C-terminal truncation of FgBlm10 prevented the degradation of Cpk1 in the *pkr* mutant. Therefore, these mutations interrupt the interaction of FgBlm10 with the 20S core proteasome and result in the dysfunction of the 26S proteasome in Cpk1 degradation. However, it is unclear whether the dysfunction of the 26S proteasome is due to the FgBlm10-mediated opening of the entrance pore or due to the FgBlm10-mediated proteasome assembly.

PKA-dependent proteasome phosphorylation is a positive regulator of 26S proteasome function [70]. When intracellular cAMP levels are increased, PKA phosphorylates the 19S subunit Rpn6 and promotes the association between 19S/RP and 20 proteasomes, and the formation of doubly capped 19S-20S-19S proteasome, hence accelerating protein degradation [71,72]. PKA also has been reported to phosphorylate the 19S/RP ATPase subunit Rpt6 [73,74,75]. In our study, FgBlm10 interacted with the majority of 19S/RP components in the presence of PKA. It is possible that phosphorylation of the subunits of the proteasome, especially those in the 19S/RP complex, facilitates the formation of the hybrid FgBlm10-CP-RP proteasome. In the absence of Pkr, the catalytic subunits of PKA were degraded, which abolished the specific phosphorylation of the 19S/RP complex by PKA and promoted the formation of the FgBlm10-CP proteasome. Nevertheless, it is also possible the phosphorylation of FgBlm10 is involved in the formation of FgBlm10-mediated proteasome. More phosphorylation sites of FgBlm10 were identified by mass spectrometry in the presence of PKA than in the *pkr* mutant, although the direct interaction of FgBlm10 and Cpk1 was not detected by pull-down assay or yeast two-hybrid assay. It is possible that FgBlm10 is phosphorylated by other kinases, which are activated by the PKA pathway.

In summary, phosphorylation of FgBlm10 indirectly by PKA or PKA-mediated phosphorylation of 19S/RP components promotes the interaction of FgBlm10 with the RP proteasome to form the FgBlm10-20S-RP hybrid proteasome, which maintains the homeostasis of catalytic subunits under the protection of Pkr in wild type PH-1. In the *pkr* mutant, FgBlm10 interacts with the 20S core proteasome and functions as the activator, and promotes the degradation of Cpk1. Non-phosphorylated proteasome components may produce a global defect in proteasome activity since the *pkr* mutant appeared to undergo rapid cell death [36]. Suppressor mutations in activator FgBlm10 or in core proteasome FgPre5 and Pre6 block their association to form the FgBlm10-20S proteasome, which prevents the degradation of Cpk1 and rescues the PKA activity. Deletion of the FgBlm10 C-terminus in the *pkr* mutant rescues the cell viability, indicating that FgBlm10 may not be involved in general protein degradation, but rather have a more specific role. Importantly, our results add a new substrate to the palette of Blm10-proteasome substrates.

## 4. Materials and Methods

### 4.1. Strains and Culture Conditions

The wild-type strain PH-1 and all the mutants of *F. graminearum* generated in this study were routinely cultured on potato dextrose agar (PDA) at 25 °C and assayed for growth rate as described [76]. Conidiation and conidium morphology were assayed with conidia harvested from 5-day-old liquid carboxymethyl cellulose (CMC) cultures [77]. Sexual reproduction and perithecium formation were examined on carrot agar cultures after self-fertilization as described [78]. For transformation of *F. graminearum*, protoplast preparation and PEG-mediated transformation were performed as described [79]. For transformant selection, hygromycin B and geneticin (Coolaber, Beijing, China) were added to the final concentration of 300 and 200 μg mL^−1^ to both top and bottom agar for selection.

### 4.2. Generation of the Fgblm10 and FgBLM10^ΔCT^ Deletion Mutants

To generate *Fgblm10* deletion mutant with the split marker approach, the flanking sequences of *FgBLM10* were amplified from PH-1 and ligated to fragments of the neomycin resistance marker amplified from pFL2 [80] by overlapping PCR. The resulting *FgBLM10* gene replacement construct was transformed into protoplasts of PH-1. Geneticin-resistant transformants were screened by PCR for the deletion of *FgBLM10*. The same approach was used to generate the *FgBLM10*^∆CT1566^ and *FgBLM10*^ΔCT11^ mutant, in which the C-terminal 1566 aa and 11 aa residues of *FgBLM10* (instead of the entire gene) were deleted.

To generate the *Fgblm10 pkr* and *FgBLM10*^∆CT1566^ *pkr* double mutants, the flanking sequences of *PKR* were amplified and ligated to the hygromycin phosphotransferase (*hph*) cassette [36] and transformed into the *Fgblm10* and *FgBLM10*^∆CT1566^ mutants. Transformants resistant to both hygromycin and geneticin were screened for the deletion of *PKR* and verified for the deletion of FgBLM10 or its CT region. All the primers used to generate and identify these mutants are listed in Table S4.

### 4.3. Generation FgPRE5^K62E^ and FgPRE6^D82N^ Allele and Transformants

The K62E mutation in FgPRE5 was introduced by overlapping PCR as described [26] with the primers listed in Table S4. The resulting PCR products were cloned into vector pKNTG carrying the geneticin-resistance marker [81]. The FgPRE5^K62E^ construct was then transformed into protoplasts of PH-1. Transformants were screened by PCR to identify FgPRE5^K62E^ transformants and verified by sequencing analysis for the K62E mutation. The FgPRE6^D82N^ transformants were generated using the same approach as described above.

### 4.4. Plant Infection Assays

Flowering wheat heads of 6-week-old wheat cultivar Xiaoyan 22 were inoculated with 10 μL of conidia suspensions (2 × 10^4^ conidia mL^−1^) at the fifth spikelet from the base as described [82]. Spikelets with typical wheat scab symptoms were examined at 14 days post-inoculation (dpi) to estimate the disease index [83]. The mean and standard deviation of the disease index was estimated with data from three independent replicates with at least ten wheat heads examined in each replicate. For assaying infectious growth, infected rachis tissues were embedded in Spurr resin after fixation and dehydration, as described previously [42]. Thick sections were then prepared and stained with 0.5% (*w*/*v*) toluidine blue as described [84]. For assaying infection cushion formation, infected lemmas were fixed with 4% (*v*/*v*) glutaraldehyde and dehydrated in a series of acetone. The samples were coated with gold–palladium and examined with a JEOL 6360 scanning electron microscope (Jeol Ltd., Tokyo, Japan) as described [84].

### 4.5. Assays for PKA Activity and Cpk1 Expression

Vegetative hyphae were harvested from 24 h YEPD (Yeast Extract Peptone Dextrose) cultures by filtration through two layers of Miracloth (Sigma, Burlington, MA, USA) and washed with double-distilled water (DDW) [85]. PKA activities were assayed with the PKA kinase assay kits (Type I) (IMMUNECHEM, Burnaby, BC, Canada) [86]. The primary antibody to *F. graminearum* Cpk1 was generated in rabbits using the synthetic oligopeptide C-VKAGAGDASQFDRYPE (ABclonal, Wuhan, China). The specificity of the resulting anti-Cpk1 antibody was verified by Western blot analysis with total proteins isolated from the *cpk1* mutant [42] and PH-1 as described [87,88]. Detection with an anti-Tub2 β-tubulin antibody [89] was used as the loading control.

### 4.6. qRT-PCR Analysis

The 12-h germlings of the wild-type strain PH-1, *Fgblm10*, *pkr*, and *Fgblm10*^∆CT1566^ mutant were collected to isolate RNA (two biological replicates each). The RNA sample was isolated by Eastep Super Total RNA Extraction Kit (Promega, Madison, WI, USA). Reverse transcription RNA used the HiScript II One Step qRT-PCR Probe Kit (Vazyme, Nanjing, China). The qRT-PCR test used the ChamQ SYBR qPCR Master Mix (Vazyme, China).

### 4.7. Affinity Purification and Mass Spectrometry Analysis

The full-length *FgBLM10* fragments were amplified with primers listed in Table S4 and cloned into vector pKNTG by one-step cloning. The GFP-FgBLM10 fusion constructs were transformed into the PH-1 and *pkr* mutant, respectively. Hyphae of the GFP-FgBLM10 transformants were extracted as described [90]. Proteins eluted from anti-GFP resins (Smart-Lifesciences, Changzhou, China) were digested with trypsin, and the resulting tryptic peptides were analyzed by nanoflow liquid chromatography-tandem mass spectrometry (MS), as described [91,92,93,94]. Proteins were identified by searching MS data against the NCBI non-redundant *F. graminearum* protein database with the SEQUEST™ algorithm [95].

### 4.8. Co-Immunoprecipitation (co-IP) Assays

The one-step cloning approach was used to generate the GFP and HIS fusion constructs. To generate the FgBLM10, and FgBLM10^∆CT^-GFP constructs, each gene was amplified and cloned into vector pKNTG [81]. FgPRE5 and FgPRE6 were also cloned into vector pKNTG to generate the HIS construct, which used a different cloning site. All of the resulting GFP and HIS fusion constructs were confirmed by sequence analysis and transformed into PH-1 in pairs. Total proteins were isolated from the resulting transformants and incubated with the anti-GFP Antibody Agarose beads (Smart-Lifesciences, China). Proteins bound to anti-GFP agarose were eluted and used for Western blot analysis [90]. The presence of related fusion proteins was detected with the anti-HIS (Cat#CW0286, CWBIO, Beijing, China) or anti-GFP (Cat #11814460001, Roche, Indianapolis, IN, USA) antibody as described [90].

### 4.9. Yeast Two-Hybrid Assays

The Matchmaker yeast two-hybrid system (Clontech, Mountain View, CA, USA) was used to assay protein-protein interactions. The FgBLM10 ORFs were amplified from the 1st-strand cDNA synthesized with the HiScript II Q RT SuperMix (Vazyme Biotech, Nanjing, China) as described [96] and cloned into pGBK7 as the bait vector. The prey constructs of CPK1 and PKR were generated with pGADT7 (Clontech). The resulting bait and prey vectors were co-transformed in pairs into yeast strain AH109 (Clontech). The Leu+ Trp+ transformants were isolated and assayed for growth on SD-Trp-Leu-His medium and galactosidase activities with filter lift assays [97]. The positive and negative controls were provided in the Matchmaker library construction kit (Clontech).

## Figures and Tables

**Table 1:** Mutations identified in the ORFs of predicted genes in suppressors of *pkr*.

Suppressor Strain	Predicted Gene	Yeast Ortholog	Mutations	
			DNA	Protein
***FgPRE5*, *FgPRE6*, and *FgBLM10* mutations identified by whole genome sequencing analysis**				
H4	FGRRES_07282	*PRE6*	G^410^ to A	D82N
H6	FGRRES_05222	*PRE5*	A^325^ to G	K62E
H10	FGRRES_16648	*BLM10*	ΔT^1220^-C^3362^	S392fs
H11	FGRRES_16648	*BLM10*	ΔG^4185^	G1380fs
	FGRRES_00324	*SNT1*	Insertion of a T after C^6065^	A1958fs
**Mutations identified by amplifying and sequencing *FgBLM10***				
H9	FGRRES_16648	*BLM10*	C^986^ to T^986^	Q314 *
H25	FGRRES_16648	*BLM10*	ΔC^997^	L317fs
H13	FGRRES_16648	*BLM10*	ΔT^1220^-C^3362^	S392fs
H23	FGRRES_16648	*BLM10*	ΔC^1234^	F396fs
H47	FGRRES_16648	*BLM10*	ΔG^2327^	N799fs
H5	FGRRES_16648	*BLM10*	C^3044^ to T^3044^	Q1000 *
H15	FGRRES_16648	*BLM10*	ΔC^3631^	L1195fs
H32	FGRRES_16648	*BLM10*	ΔC^3631^	L1195fs
H21	FGRRES_16648	*BLM10*	ΔC^3631^	L1195fs

*, Stop codon fs; frameshift mutant.

**Table 2:** The wild-type and mutant strains of *F. graminearum* used in this study.

Strains	Brief Description	References
PH-1	Wild type	[43]
*pkr*	*pkr* deletion mutant of PH-1	[36]
H4, H6, H10, H11, H5, H9, H13, H15, H21, H23, H25, H32, H47	Spontaneous suppressor mutants of *pkr* mutant	[36]
BM1	*Fgblm10* deletion mutant of PH-1	This study
BM5	*Fgblm10* deletion mutant of PH-1	This study
BM13	*Fgblm10* deletion mutant of PH-1	This study
BN3	*FgBLM10*^∆CT1566^ mutant of PH-1	This study
BN6	*FgBLM10*^∆CT1566^ mutant of PH-1	This study
BN9	*FgBLM10*^∆CT1566^ mutant of PH-1	This study
BNP2	*Fgblm10 pkr* mutant of PH-1	This study
BNP5	*Fgblm10 pkr* mutant of PH-1	This study
BNP10	*Fgblm10 pkr* mutant of PH-1	This study
BNP1	*FgBLM10*^∆CT1566^ *pkr* mutant of PH-1	This study
BNP4	*FgBLM10*^∆CT1566^*pkr* mutant of PH-1	This study
BNP10	*FgBLM10*^∆CT1566^*pkr* mutant of PH-1	This study
BNYP4	*FgBLM10*^∆CT11^*pkr* mutant of PH-1	This study
BNYP10	*FgBLM10*^∆CT11^*pkr* mutant of PH-1	This study
BPR5-2	*FgBLM10*-GFP and *FgPRE5*-His transformant of PH-1	This study
BPR5-1	*FgBLM10*-GFP and *FgPRE5*-His transformant of PH-1	This study
BPR5-4	*FgBLM10*-GFP and *FgPRE5*-His transformant of PH-1	This study
BPR6-1	*FgBLM10*-GFP and *FgPRE6*-His transformant of PH-1	This study
BPR6-3	*FgBLM10*-GFP and *FgPRE6*-His transformant of PH-1	This study
BPEK3	*FgBLM10*-GFP and *FgPRE5*^K62E^-His transformant of PH-1	This study
BPEK4	*FgBLM10*-GFP and *FgPRE5*^K62E^-His transformant of PH-1	This study
BPRD5	*FgBLM10*-GFP with *FgPRE6*^D82N^ -His transformant of PH-1	This study
BPRD7	*FgBLM10*-GFP with *FgPRE6*^D82N^ -His transformant of PH-1	This study
BCPR6-1	*FgBLM10*^∆CT1566^-GFP and *FgPRE6*-His transformant of PH-1	This study
BCPR6-3	*FgBLM10*^∆CT1566^-GFP and *FgPRE6*-His transformant of PH-1	This study
BCPR5-3	*FgBLM10*^∆CT1566^-GFP and *FgPRE5*-His transformant of PH-1	This study
BCPR5-4	*FgBLM10*^∆CT1566^-GFP and *FgPRE5*-His transformant of PH-1	This study

**Table 3:** Phenotypes of the *FgBlm10* mutant and its transformant strains in growth, conidiation, and plant infection.

Strain	Growth Rate(mm/Day) ^a,b^	Conidiation (×10^4^ Conidia/mL) ^a,c^	Disease Index ^a,d^
PH-1	10.5 ± 0.3 ^a^	247.6 ± 24.7 ^a^	9.8 ± 2.6 ^a^
*pkr*	2.9 ± 0.4 ^f^	14.2 ± 2.6 ^de^	0 ± 0 ^d^
*Fgblm10* ^∆CT11^ *pkr*	8.3 ± 0.6 ^b^	10.2 ± 5.6 ^bc^	0 ± 0 ^d^
*Fgblm10*	8.4 ± 0.4 ^b^	97.9 ± 15.9 ^b^	6.5 ± 1.5 ^b^
*Fgblm10* ^∆CT1566^	7.5 ± 0.2 ^d^	140.2 ± 23.9 ^a^	7.7 ± 1.5 ^b^
*Fgblm10 pkr*	3.2 ± 0.5 ^f^	46.3 ± 9.3 ^c^	0.3 ± 0.5 ^c^
*Fgblm10* ^∆CT1566^ *pkr*	7.9 ± 0.4 ^c^	49.6 ± 11.1 ^c^	1.0 ± 0.0 ^c^

^a^ Standard deviation (mean ± standard deviation) were calculated from at least three independent measurements; ^b^ Average daily extension of colony radium; ^c^ Conidiation was measured with 5-day-old Carboxymethylcellulose (CMC) culture; ^d^ Diseased spikelets per wheat head examined 14 dpi; Different letters indicate significant differences based on ANOVA analysis followed by Duncan’s multiple range test (*p =* 0.05) in a, b, c, d, e, f.

**Table 4:** Proteasome-associated proteins co-immunoprecipitated with FgBlm10-GFP in the wild type and *pkr* mutant.

Genes	Homologs in *S. cerevisiae*	Function	PSMs	
			PH-1	*pkr*
FGRRES_16648	Blm10		1341	469
20S core particle				
FGRRES_07282_M	PRE6	α4	1	1
FGRRES_10255_M	SCL1	α1	2	0
FGRRES_04410	PRE8	α2	2	0
FGRRES_05365	PRE9	α3	1	0
FGRRES_01160	PRE7	β6	1	0
19S regulatory particle				
FGRRES_07956	RPN9	Structural	21	5
FGRRES_08444	RPN7	Structural	12	3
FGRRES_01198	RPT4	ATPase	12	4
FGRRES_01605	RPT6	ATPase	14	2
FGRRES_00306	RPN6	Structural	19	0
FGRRES_09432	RPN5	Structural	18	0
FGRRES_06045_M	RPN3	Structural	12	0
FGRRES_07938	RPN12	Structural	12	0
FGRRES_10738	RPN8	Structural	7	0
FGRRES_16839	RPN2	Structural	16	0
FGRRES_00559_M	RPT1	ATPase	10	0
FGRRES_02028	RPT2	ATPase	9	0
FGRRES_10769	RPT3	ATPase	12	0
FGRRES_11597	RPT5	ATPase	12	0
FGRRES_09783	RPN1	Ubp6 and ubiquitin binding	17	0
FGRRES_00781	RPN11	Deubiquitinase	8	0
FGRRES_01123	RPN10	Ubiquitin binding	3	0
FGRRES_10724	-	26S proteasome regulatory subunit n6	1	0

- No homolog.

**Table 5:** Growth phenotypes of the *FgBlm10* mutant and its transformant strains treated with bleomycin.

Strains	Growth Rate (mm/Day) ^a,b^
	Bleomycin
PH-1	4.06 ± 0.08 ^c^
*pkr*	1.52 ± 0.10 ^e^
*Fgblm10*	4.50 ± 0.30 ^b^
*FgBLM10* ^∆CT1566^	5.39 ± 0.05 ^a^
*Fgblm10 pkr*	0.00 ± 0.00 ^f^
*FgBLM10* ^∆CT1566^ *pkr*	2.81 ± 0.16 ^d^

^a^ Standard deviation (mean ± standard deviation) was calculated from at least three independent measurements; ^b^ Average daily extension of colony radius; Different letters indicate significant differences based on ANOVA analysis followed by Duncan’s multiple range test (*p = 0.05*) in a, b, c, d, e, f.

## Data Availability

The genomic sequence data were deposited in the NCBI GEO repository (accession number PRJNA855364).

## Supplementary Materials

The following supporting information can be downloaded at: https://www.mdpi.com/article/10.3390/ijms231810208/s1. Reference [98] is cited in the supplementary materials.
