# Supplementary material for: PKR Protects the Major Catalytic Subunit of PKA Cpk1 from FgBlm10-Mediated Proteasome Degradation in Fusarium graminearum
Source: Int J Mol Sci. 2022 Sep 6;23(18):10208. doi: 10.3390/ijms231810208 (PMC9499325; doi:10.3390/ijms231810208)
Supplement: Supplementary file 1 [file ijms-23-10208-s001.zip › Table S1 mutant sites.pdf]

Table S1 Mutations identified in the *pkr* suppressor strains

	**Fg00324**	**Fg01210**	**Fg07282**	**Fg05222**	**Fg09715**	**Fg12901**	**Fg05396**	**Fg00385**	**Fg04083**	**Fg03942**	**Fg10588**	**Fg10589**	**Fg00887**
Sc	*SNT1*	*BLM10*	*PRE6*	*PRE5*	*MIG1*	*CYC8*	*SGF73*	*NHP6A/6B*	NA	NA	NA	NA	NA
Sp	*SNT1*	NA	*PRE6*	*PRE5/*	*SCR1*	*SSN6*	*SGF73*	*NHP6*	NA	NA	NA	NA	NA
Human	*NCOR1*	*PSME4*	*PSMA7*	*PSMA1*	NA	*KDM6A*	*ATXN7*	*HMGB1*	NA	NA	NA	NA	NA
Mutations identified by whole genome sequencing analysis													
H4	-	-	D82N	-	-	-	-	-	-	-	-	-	-
H6	-	-	-	K62E	-	-	-	-	-	-	-	-	-
H10	-	S392fs	-	-	-	-	-	-	-	-	-	-	-
H11	A1958fs	G1380fs	-	-	-	-	-	-	-	-	-	-	-
H17	Y2135fs	-	-	-	-	-	-	-	-	-	-	-	-
H22	-	-	-		-	G88D	-	-	-	-	del	del	del
H28	-	-	-	-	-	-	-	-	-	R140M	-	-	-
H30	-	-	-	-	-	-	-	L57P	M368fs	-	-	-	-
H35							R257K						
H57	-	-	-	-	K71E	-	-	-	-	-	-	-	-
Mutations identified by amplifying and sequencing the interested genes													
H1	-	-	-	-	-	-	-	-	NS	NS	NS	NS	NS
H2	-	-	-	-	-	-	-	-	NS	NS	NS	NS	NS
H3	-	-	-	-	-	-	-	-	NS	NS	NS	NS	NS
H5	A1958fs	Q1000*	-	-	-	-	-	-	NS	NS	NS	NS	NS
H7	A1958fs	-	-	-	-	-	-	-	NS	NS	NS	NS	NS
H8	-	-	-	-	-	-	-	-	NS	NS	NS	NS	NS
H9	-	Q314*	-	-	-	-	-	-	NS	NS	NS	NS	NS
H12	-	-	-	-	-	-	-	-	NS	NS	NS	NS	NS
H13	-	S392fs	-	-	-	-	-	-	NS	NS	NS	NS	NS
H14	-	-	-	-	-	-	-	-	NS	NS	NS	NS	NS
H15	-	L1195fs	-	-	-	-	-	-	NS	NS	NS	NS	NS
H16	Y2135fs	-	-		-	-	-	-	NS	NS	NS	NS	NS
H18	-	-	-	-	-	-	-	-	NS	NS	NS	NS	NS
H19	-	-	-	-	-	-	-	-	NS	NS	NS	NS	NS
H20		-	-	-	-	-	-	-	NS	NS	NS	NS	NS
H21	-	L1195fs	-	-	-	-	-	-	NS	NS	NS	NS	NS
H23	-	F396fs	-	-	-	-	-	-	NS	NS	NS	NS	NS
H24	-	-	-	-	-	G88D	-	-	NS	NS	NS	NS	NS
H25	-	L317fs	-	-	-	-	-	-	NS	NS	NS	NS	NS
H26	Y2135fs	-	-	-	-	-	-	-	NS	NS	NS	NS	NS
H27	-	-	-	-	-	-	-	-	NS	NS	NS	NS	NS
H29	Y2135fs	-	-	-	-	-	-	-	NS	NS	NS	NS	NS
H31	-	-	-	-	-	-	-	-	NS	NS	NS	NS	NS
H32	-	L1195fs	-	-	-	-	-	-	NS	NS	NS	NS	NS
H33	-	-	-	-	-	-	-	-	NS	NS	NS	NS	NS
H34	-	-	-	-	-	-	-	-	NS	NS	NS	NS	NS
H36	-	-	-	-	-	-	-	-	NS	NS	NS	NS	NS
H37	-	-	-	-	-	-	-	-	NS	NS	NS	NS	NS
H38	-	-	-	-	-	-	-	-	NS	NS	NS	NS	NS
H39	-	-	-	-	-	-	-	-	NS	NS	NS	NS	NS
H40	-	-	-	-	-	-	-	-	NS	NS	NS	NS	NS
H41	-	-	-	-	-	-	-	-	NS	NS	NS	NS	NS
H42	-	-	-	-	-	-	-	-	NS	NS	NS	NS	NS
H43	-	-	-	-	-	-	-	-	NS	NS	NS	NS	NS
H44	Y2135fs	-	-	-	-	-	-	-	NS	NS	NS	NS	NS
H45	-	-	-	-	-	-	-	-	NS	NS	NS	NS	NS
H46	-	-	-	-	-	-	-	-	NS	NS	NS	NS	NS
H47	-	N799fs	-	-	-	-	-	-	NS	NS	NS	NS	NS
H48	-	-	-	-	-	-	-	-	NS	NS	NS	NS	NS
H49	-	-	-	-	-	-	-	-	NS	NS	NS	NS	NS
H50	-	-	-	-	-	-	-	-	NS	NS	NS	NS	NS
H51	Y2135fs	-	-	-	-	-	-	-	NS	NS	NS	NS	NS
H52	Y2135fs	-	-	-	-	-	-	-	NS	NS	NS	NS	NS
H53	Y2135fs	-	-	-	-	-	-	-	NS	NS	NS	NS	NS
H54	-	-	-	-	-	-	-	-	NS	NS	NS	NS	NS
H55	-	-	-	-	-	-	-	-	NS	NS	NS	NS	NS
H56	-	-	-	-	-	-	-	-	NS	NS	NS	NS	NS
H58	-	-	-	-	-	-	-	-	NS	NS	NS	NS	NS
H59	-	-	-	-	L291fs	-	-	-	NS	NS	NS	NS	NS
H60	-	-	-	-	-	-	-	-	NS	NS	NS	NS	NS

-, sequenced but no changes found
*, stop codon
fs, frameshift mutation
del, deletion of the whole open reading frame
NS, not sequenced
