# Supplementary material for: PKR Protects the Major Catalytic Subunit of PKA Cpk1 from FgBlm10-Mediated Proteasome Degradation in Fusarium graminearum
Source: Int J Mol Sci. 2022 Sep 6;23(18):10208. doi: 10.3390/ijms231810208 (PMC9499325; doi:10.3390/ijms231810208)
Supplement: Supplementary file 1 [file ijms-23-10208-s001.zip › Table S3 Proteins coimmunoprecipitated with FgBlm10.pdf]

Table S3 Proteins coimmunoprecipitated with FgBlm10-GFP in a pulldown assay.

Accession	Description	PSMs in PH-1	*PSMs in pkr*
FGRRES_16648	Blm10	1341	469
**proteasome**			
FGRRES_07282_M	20S proteasome component pre6	1	1
FGRRES_10255_M	20S proteasome subunit alpha 1	2	0
FGRRES_04410	20S proteasome subunit alpha type-2	2	0
FGRRES_05365	20S proteasome subunit alpha type-4	1	0
FGRRES_01160	20S proteasome subunit beta 6	1	0
FGRRES_07956	26s proteasome regulatory subunit n9	21	5
FGRRES_08444	26s proteasome regulatory subunit n7	12	3
FGRRES_00306	26s proteasome regulatory subunit n6	19	0
FGRRES_09432	26s proteasome regulatory subunit n5	18	0
FGRRES_06045_M	26s proteasome regulatory subunit n3	12	0
FGRRES_07938	26s proteasome regulatory subunit n12	12	0
FGRRES_00781	26s proteasome regulatory subunit rpn11	8	0
FGRRES_10738	26s proteasome regulatory subunit rpn-8	7	0
FGRRES_01123	26s proteasome regulatory subunit n10	3	0
FGRRES_00559_M	26s protease regulatory subunit 7	10	0
FGRRES_02028	26s proteasome regulatory subunit t2	9	0
FGRRES_10769	26s protease regulatory subunit 6b	12	0
FGRRES_01198	26s protease subunit rpt4	12	4
FGRRES_11597	26s protease regulatory subunit 6a	12	0
FGRRES_01605	26s protease regulatory subunit 8	14	2
FGRRES_09783	26s proteasome regulatory subunit rpn-1	17	0
FGRRES_16839	26s proteasome regulatory subunit n2	16	0
**Ubiquitin system**			
FGRRES_00380	ubiquitin carboxyl-terminal hydrolase 16	1	0
FGRRES_01863	ubiquitin carboxyl-terminal hydrolase l5	1	0
FGRRES_15861	ubiquitin conjugation factor e4 b	3	0
FGRRES_06244	ubiquitin supergroup	1	0
FGRRES_10925	ubiquitin thiolesterase	17	0
FGRRES_08920	ubiquitin thiolesterase	6	0
FGRRES_16824	ubiquitin thiolesterase	6	0
FGRRES_10292	ubiquitin thiolesterase	4	0
FGRRES_06789	ubiquitin thiolesterase	1	0
FGRRES_10941	ubiquitin-40s ribosomal protein s27a	17	0
FGRRES_09689	ubiquitin-activating enzyme e1	12	0
FGRRES_01505	ubiquitin-conjugating enzyme e2 c	1	0
FGRRES_04032	ubiquitin-conjugating enzyme e2 d e	1	0
FGRRES_09630	ubiquitin-conjugating enzyme e2 g1	2	0
FGRRES_05134	ubiquitin-conjugating enzyme e2 r	1	0
FGRRES_10805	ubiquitin-conjugating enzyme e2-16 kda	2	0
FGRRES_10226	ubiquitin-like modifier-activating enzyme	1	0

	atg-7		
FGRRES_04300	ubl-specific protease	1	0
**Nuclear import**			
FGRRES_07467	nucleosome assembly protein 1-like 1	2	0
FGRRES_05972	nucleoside-diphosphate kinase	3	0
FGRRES_01429	nucleoporin-interacting protein nic96	2	0
FGRRES_05487	nucleoporin nup82	1	0
FGRRES_11878_M	nucleolin	5	0
FGRRES_10908	nucleolar protein nop58-like protein	22	0
FGRRES_02511	nucleolar protein nop4	5	0
FGRRES_07038	nucleolar protein 9	2	0
FGRRES_06243	nucleolar protein	6	0
FGRRES_06165	nucleolar gtp-binding protein	9	0
FGRRES_00292	nucleolar complex protein 2	10	0
FGRRES_17145	nuclear protein snf4	1	0
FGRRES_01216	nuclear protein localization protein 4	3	0
FGRRES_05671	nuclear pore protein	5	0
FGRRES_09965	nuclear pore protein	4	0
FGRRES_01323	nuclear pore protein	2	0
FGRRES_09165	nuclear movement protein nudc	3	0
FGRRES_01185	nuclear gtp-binding protein nug1	10	0
FGRRES_10865	nuclear envelope protein ndc1	1	0
FGRRES_05038	nuclear distribution protein pac1	1	0
FGRRES_11878_M	nucleolin	0	2
**DNA repair**			
FGRRES_09801	dna topoisomerase ii	1	
FGRRES_09663	dna topoisomerase 2-associated protein pat1	6	
FGRRES_05599	dna repair transcription protein mms19	4	
FGRRES_01052	proliferating cell nuclear antigen	1	0
FGRRES_16593	mortality factor 4-like protein 1	3	
