# Supplementary material for: PKR Protects the Major Catalytic Subunit of PKA Cpk1 from FgBlm10-Mediated Proteasome Degradation in Fusarium graminearum
Source: Int J Mol Sci. 2022 Sep 6;23(18):10208. doi: 10.3390/ijms231810208 (PMC9499325; doi:10.3390/ijms231810208)
Supplement: Supplementary file 1 [file ijms-23-10208-s001.zip › Table S4 primer.pdf]

Table S4

PCR primers used in this study

Name	Sequence(5’-3’)
BLM10-Seq-1F	ATGGACGAGAACATCGGACCT
BLM10- Seq-1R	GCAAGAACAAGGAGTGGTCA
BLM10- Seq-2F	TTTCCCCAATCATCGACG
BLM10- Seq-2R	GGCAAAGTAACTCTTCCAGAGTACTCC
PRE5- Seq-F	ATGTTTCGAAACAACTACGATAACGATTC
PRE5- Seq-R	TTAGCTGTCAACGTCCATACCTTC
PRE6- Seq-F	ATGGTGGATCCCAGCTCCG
PRE6- Seq-R	TTATTCCGCAGCAGAGTCATCTTGA
BLM10-1F	CAGGTAGCTTCCATACATAGTAGCATA
BLM10-2R	CAGATACGGCAGAGAAATCGCAACCTCAGTGGACAGATGATGTGTGTG
BLM10-3F	GTTTAGATTCCAAGTGTCTACTGCTGGCTGGAAGCGAATCAGGCAGGGC
BLM10-4R	GACATTATTCTGATACGATCAACGC
BLM10-5F	TTTGTATGAGCCTGAGGTCATTCAG
BLM10-6R	CTTGTCCAACTCGCTCTTCTG
BLM10-7F	GCTCAATATAGCACCGAGAACT
BLM10-8R	CTTTGAACCAATCACAAGGCT
HYG-F	GGCTTGGCTGGAGCTAGTGGAGGTCAA
HY-R	GTATTGACCGATTCCTTGCGGTCCGAA
YG-F	GATGTAGGAGGGCGTGGATATGTCCT
HT-R	GTCGACTTAATAACACATTGCGGACGT
H855F	GTCGATGCGACGCAATCGT
HT856R	ATCAGCCCCACTTGTAGCAGTAGC
$BLM10^{N392-}3F$	GTTTAGATTCCAAGTGTCTACTGCTGGCAGACTCCATGAATCCCTCCAAATGA
PKR-1F	TGTGCGTCAACTATGGGTCTGG
PKR-2R	AATGCTCCTTCAATATCATCTTCTGTCAGATGTTATGTCGTCGAAGAGGC
PKR-3F	CGTCCGCAATGTGTTATTAAGTCGACAGTGATGTTCAGGTTGTCTCGTG
PKR-4R	AGCCAGGAGAAATAACGCACC
PKR-5F	ACGACACCATCACATCGCCTAC
PKR-6R	AGATCCCTTCTCGACGACATAGAA
PKR-7F	GCCTTGTGAGCCCTTGTTGTG
PKR-8R	GGAAACCGAAGTTGCTGTTGAGG
GEN-F	GAGGTTGCGATTTCTCTGCCGTATCTG
GEN-R	GCCAGCAGTAGACACTTGGAATCTAAAC
G852-F	TCGGCTATGACTGGGCACAACA
G850-R	GAGCGGCGATACCGTAAAGCAC

G855-R	TGTTGGGTTTGAGCTAGGTGGG
G856-F	GAATGGTCAAATCAAACTGCTAGATAT
Blm10-motif-1f	GCGCCATGAAAGAACACCG
Blm10-motif-2r:	CAGATACGGCAGAGAAATCGCAACCTCCTAATCAATTGTTAGCGA
BLM10-motif-3f	GTTTAGATTCCAAGTGTCTACTGCTGGCAGGCAGGGCTGTGTTCAG
BLM10-motif-4r	CATATCGTCACTGTTAGTAAAATACGTGTGGT
Blm10-motif-5f	TATTTTACTTCCGAACAGCT
Blm10-motif-6r	GGCAAAGTAACTCTTCCAGA
Blm10-motif-7F	TTCGACAACTGGTATCAGAC
Blm10-motif-8R	ATAAGTGTCGGGATGAATGT
PKNTG-<br>BLM10- F:	AGGAACCCAATCTTCAAACTCGAGATGTAAGGTTACAACATCATCCC<br>ACT
BLM10^{N392}-R:	TGATTTCAGTAACGTTAAGTGGATCCAATAGACTCCATGAATCCCTCC<br>AAATG
Blm10-R:	TGATTTCAGTAACGTTAAGTGGATCCGGCAAAGTAACTCTTCCAGAGT<br>ACT
PKNTG-Pre5-F:	AGGAACCCAATCTTCAAACTCGAGCATCATCACCATCACCATATGTTT<br>CGAAACAACTACGATAACGATT
Pre5-R:	TGATTTCAGTAACGTTAAGTGGATCCGCTGTCAACGTCCATACCTT
PKNTG-Pre6-F:	GAACCCAATCTTCAAACTCGAGCATCATCACCATCACCATATGGCTTC<br>CGGATACGACAG
Pre6-R:	TGATTTCAGTAACGTTAAGTGGATCCTTCCGCAGCAGAGTCATCT
Cpk1-RT-F	AGCAGTCTCAGCAACGATAC
Cpk1-RT-R	GTTCCAAGCGTCCTCAAGATA
Pre6^{D82N}-His-F:	TTAAACGCAAACGCTCGTATCCTCG
Pre6^{D82N}-His-R:	CGAGGATACGAGCGTTTGCGTTTAA
Pre5^{K62E}-His-F:	CTTACCAGAAGGAGCTCTTCACCG
Pre5^{K62E}-His-R:	CGGTGAAGAGCTCCTTCTGGTAAG
PKR-AD-F:	GCCATGGAGGCCAGTGAATTCATGTCCGGCCCTTTCTCC
PKR-AD-R:	CCACTGCTTGGGTGGAATTCGCCGGTGTGAAGTGGAT
Cpk1-AD-F	GCCATGGAGGCCAGTGAATTCGCCGGTGTGAAGTGGAT
Cpk1-AD-R	CCACTGCTTGGGTGGAATTCATGCCTTCACTGGGGTTTCT
BLM10-BD-F:	GAGGACCTGCATATGATGGACGAGAACATCGGACCT
BLM10-BD-R:	CTCCATGGCCATATG GGCAAAGTAACTCTTCCAGAGTACTCC
Blm10$^{1\text{-}392}$-AD-F	GCCATGGAGGCCAGTGAATTCATGGACGAGAACATCGGACCTCGCC
Blm10$^{1\text{-}392}$-AD-R	CCACTGCTTGGGTGGAATTCAGACTCCATGAATCCCTCCAAATGAGAT<br>AT
Blm10$^{913\text{-}1924}$-<br>BD-F	ATGGCCATGGAGGCCGAATTCATGGACCCTGAGCGTGCCGCGGGCAT
Blm10$^{913\text{-}1924}$-<br>BD-F	CGACGGATCCCCGGGAATTCCTCGCTAAGAATTGTCTTGGTCGC
