# Supplementary material for: PKR Protects the Major Catalytic Subunit of PKA Cpk1 from FgBlm10-Mediated Proteasome Degradation in Fusarium graminearum
Source: Int J Mol Sci. 2022 Sep 6;23(18):10208. doi: 10.3390/ijms231810208 (PMC9499325; doi:10.3390/ijms231810208)
Supplement: Supplementary file 1 [file ijms-23-10208-s001.zip › supplementary figures.pdf]

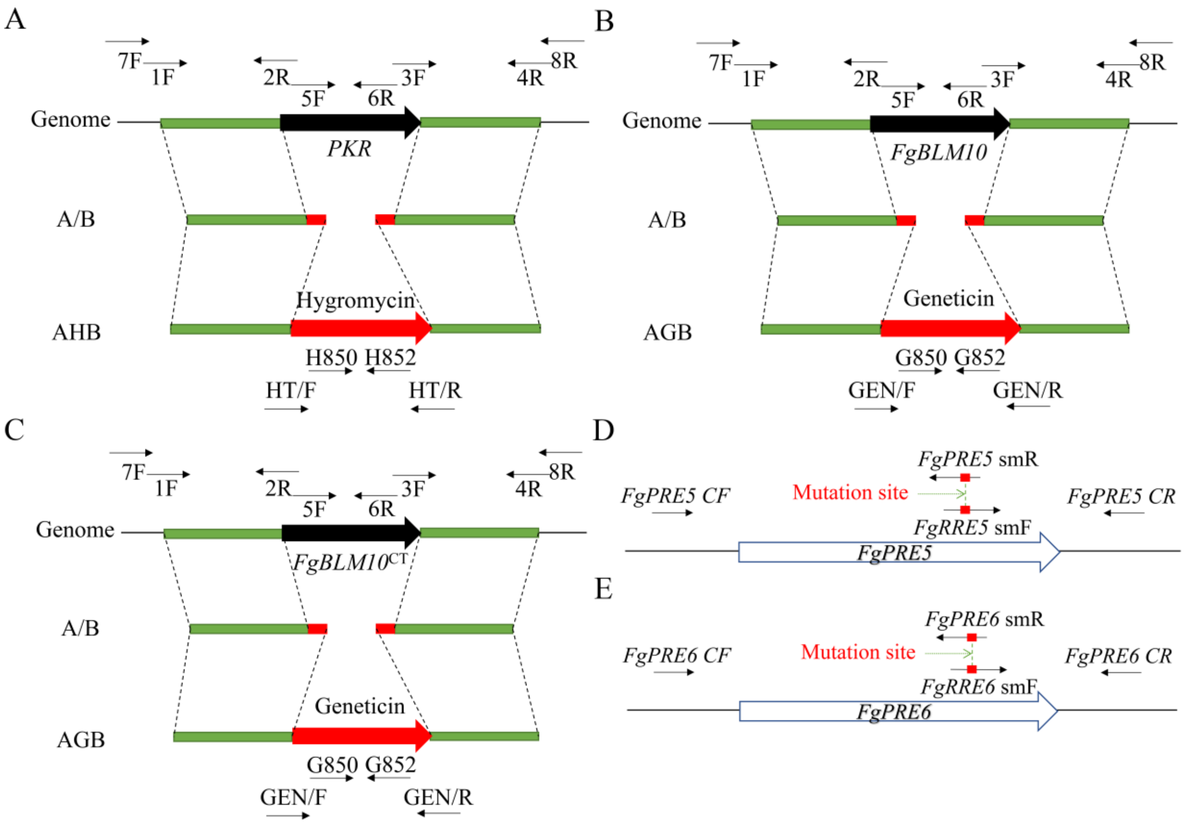

Figure S1 Schematic diagram of the primers used to generate PKR, FgBLM10 and FgBLM10ΔCT gene replacement constructs, and schematic diagram of the primers used to generate FgPre5K62E and Pre6D82N allele.

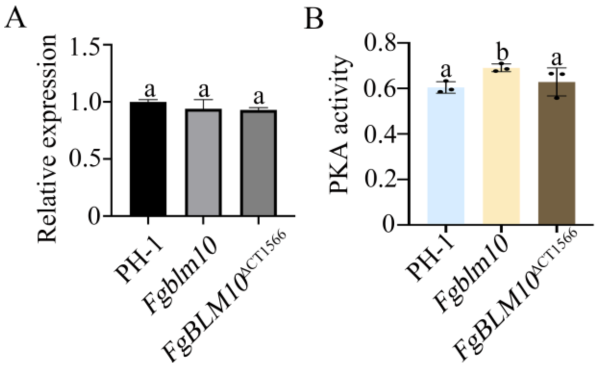

Figure S2 CPK1 expression and PKA activity in the Fgblm10 and FgBLM10ΔCT1566 mutant; (A) qRT-PCR analysis of the wild-type stain PH-1, the Fgblm10 mutant, and the FgBLM10ΔCT1566 mutant; (B) PKA activity was assayed with proteins isolated from hyphae of PH-1, the Fgblm10 mutant, and the FgBLM10ΔCT1566 mutant.

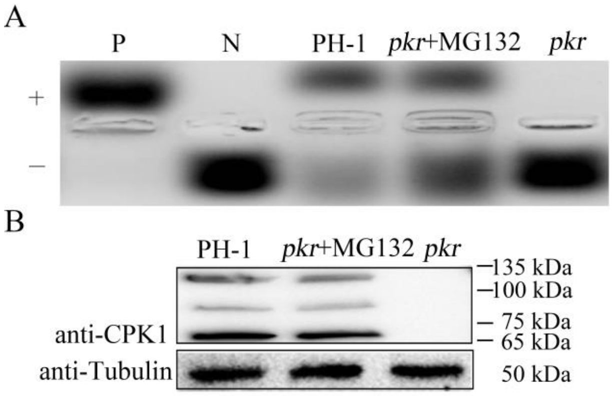

Figure S3 Assays for PKA activity and Cpk1 expression. (A) Vegetative hyphae were harvested from 24-h YEPD cultures. PKA activities were assayed with the PepTag nonradioactive PKA assay kit (Promega, Madison, WI) as described [98]. (B) Western blots of proteins isolated from the marked strains were detected with an anti-FgCpk1 antibody

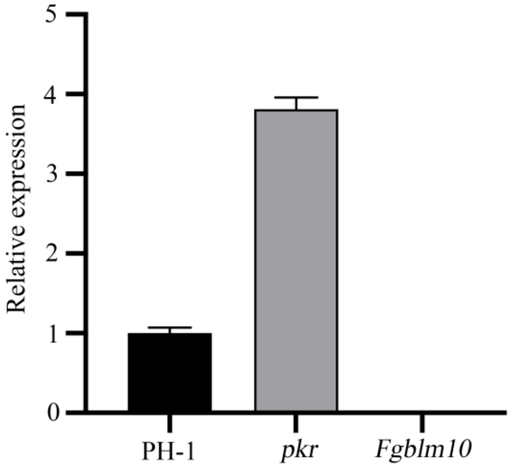

Figure S4 FgBLM10 expression in wild type strain PH-1, Fgblm10, and pkr mutant.

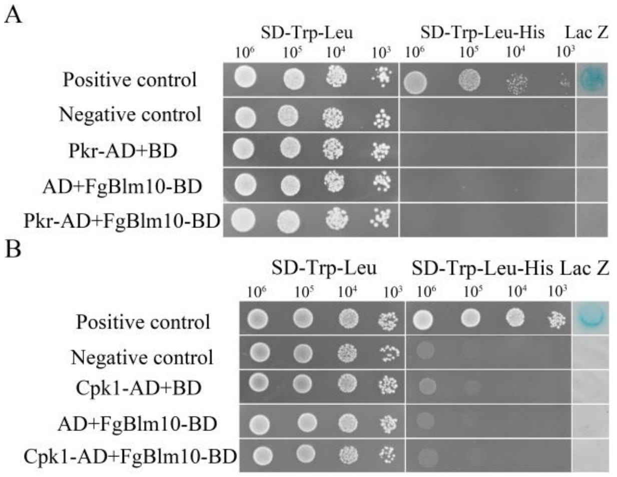

Figure S5 Yeast two-hybrid assays for the interaction of Pkr and Cpk1 (bait) with FgBlm10 or FgBlm10ΔCT1566 (prey). The positive and negative controls were from the Matchmaker kit.

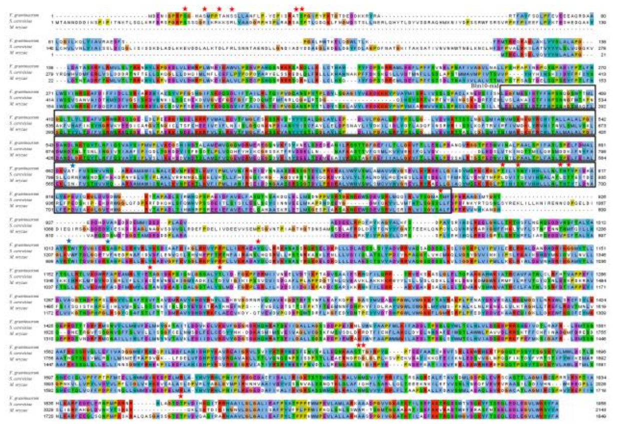

Figure S6 Schematic drawing of the FgBlm10 protein and alignment with homologs from Fusarium graminearum (Fg), Saccharomyces cerevisiae (Sc), Neurospora crassa (Nc), Magnaporthe oryzae (Mo), F. oxysporum (Fo), and F. verticillioides (Fv). Phosphorylation sites of FgBlm10 in PH-1 are indicated by red stars, and phosphorylation sites of FgBlm10 in pkr mutant are indicated by blue stars.

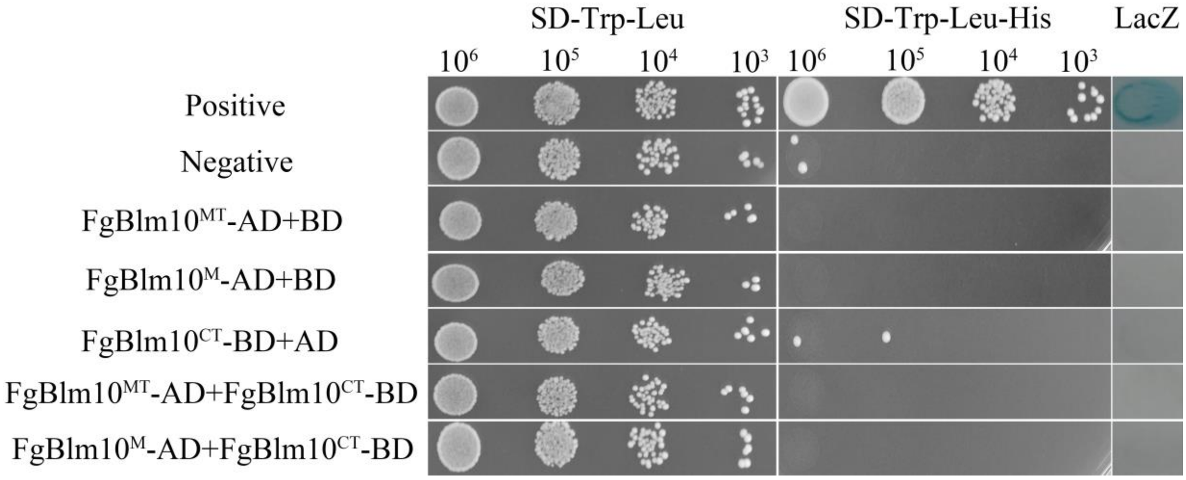

Figure S7 Yeast two-hybrid assays for the interaction of FgBlm10 N-terminus with FgBlm10 C-terminus. The positive and negative controls were from the Matchmaker kit.

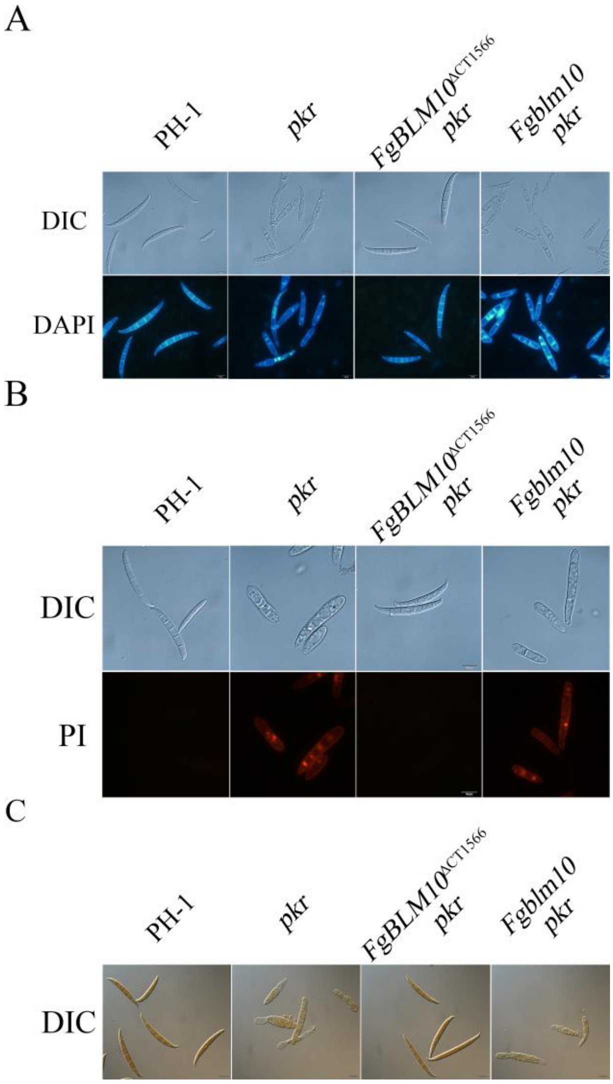

Figure S8 FgBLM10ΔCT1566 rescued the defects of the pkr mutant in cell viability, and glycogen accumulation. (A) Conidia of wild-type PH-1 (WT), pkr mutant (pkr), Fgblm10 mutant, and FgBLM10ΔCT1566 mutant harvested from 5-day-old carboxymethyl cellulose (CMC) cultures and stained with DAPI (4',6-diamidino-2-phenylindole) to indicate the living cells. Bar = 10 μm. (B) Conidia of the marked strains were stained with 5 μg/mL propidium iodide (PI) to indicate the dead cells. Bar = 10 μm. (C) Conidia of the marked strains were stained with KI/I2 solution to indicate glycogen accumulation. Bar = 10 μm.
