# Supplementary material for: PKR Protects the Major Catalytic Subunit of PKA Cpk1 from FgBlm10-Mediated Proteasome Degradation in Fusarium graminearum
Source: Int J Mol Sci. 2022 Sep 6;23(18):10208. doi: 10.3390/ijms231810208 (PMC9499325; doi:10.3390/ijms231810208)
Supplement: Supplementary file 1 [file ijms-23-10208-s001.zip › New Table S2 suppressor growth rate conidiation infection.pdf]

Table.S2 Phenotypes of suppressor strains which mutations in *FgBLM10* in growth, conidiation.

**Strain**	**Growth rate**(mm/day)[a,b]	**Conidiation** (×10^{4} conidia/mL)[a,c]
PH-1	9.7 ± 0.4[a]	153.3 ± 62.2[a]
pkr	2.2 ± 0.2[g]	13.7 ± 4.2[d]
H4	5.7 ± 0.6[e]	55.6 ± 17.5[b]
H5	6.3 ± 0.4[cd]	30.8 ± 17.3[c]
H6	6.6 ± 0.4[bcd]	2.4 ± 2.1[e]
H9	6.9 ± 0.5[b]	18.2 ± 12.3[cd]
H10	6.7 ± 0.6[bc]	23.8 ± 5.6[c]
H11	6.9 ± 0.6[b]	14.4 ± 10.1[d]
H15	6.9 ± 0.4[b]	0.8 ± 0.2[e]
H21	6.8 ± 0.3[b]	14.1 ± 7.7[d]
H23	7.1 ± 0.2[d]	0.8 ± 0.4[e]
H25	6.1 ± 0.4[de]	4.4 ± 2.5[e]
H32	6.6 ± 0.4[bcd]	2.7 ± 1.5[e]
H47	4.5 ± 1.3[f]	0 ± 0[f]

[a] Standard deviation (mean ± standard deviation) were calculated from at least three independent measurements.
[b] Average daily extension of colony radium.
[c] Conidiation was measured with 5-day-old Carboxymethylcellulose(CMC) culture.
Different letters indicate significant differences based on ANOVA analysis followed by Duncan's multiple range test ($P = 0.05$) in a, b, c, d, e, f, g.
